# Suertides A–C: selective antibacterial cyclic hexapeptides from *Amycolatopsis* sp. MST-135876v3

**DOI:** 10.1038/s41429-022-00544-4

**Published:** 2022-07-26

**Authors:** Heather J. Lacey, Rachel Chen, Daniel Vuong, Mark F. Fisher, Ernest Lacey, Peter J. Rutledge, Andrew M. Piggott

**Affiliations:** 1 Microbial Screening Technologies, Sydney, NSW 2164 Australia; 2 School of Chemistry, The University of Sydney, Sydney, NSW 2006 Australia; 3 School of Molecular and Life Sciences, Curtin University, Perth, WA 6102 Australia; 4 School of Natural Sciences, Macquarie University, Sydney, NSW 2109 Australia

**Keywords:** Bacteria, Organic chemistry

## Abstract

*Amycolatopsis* sp. MST-135876 was isolated from soil collected from the riverbank of El Pont de Suert, Catalonia, Spain. Cultivation of MST-135876 on a range of media led to the discovery of a previously unreported dichlorinated cyclic hexapeptide, suertide A (d-Ser, 5-Cl-d-Trp, 6-Cl-d-Trp, l-Ile, d-Val, d-Glu), featuring an unprecedented pair of adjacent 5/6-chlorotryptophan residues. Supplementing the growth medium with KBr resulted in production of the mono- and dibrominated analogues suertides B and C, respectively. Suertides A–C displayed selective activity against *Bacillus subtilis* (MIC 1.6 µg ml^−1^) and *Staphylococcus aureus* (MIC 3.1, 6.3, and 12.5 µg ml^−1^, respectively), while suertides A and B showed appreciable activity against methicillin-resistant *S. aureus* (MIC 1.6 and 6.3 µg ml^−1^, respectively).

## Introduction

The genus *Amycolatopsis* was first described in 1986 along with *Amycolata* to accommodate nocardioform actinomycetes, forming new branches in the evolutionary tree of Pseudonocardiaceae [1]. Before this, *Amycolatopsis* species were categorised as *Streptomyces* and then *Nocardia*. To date, 96 verified *Amycolatopsis* species have been acknowledged in the List of Prokaryotic names with Standing in Nomenclature (LPSN) database [2], making it the 28^th^ most well-described bacterial genus. A thorough review of the published *Amycolatopsis* secondary metabolites by Song et al. [3] revealed that between 1989 and 2020, 159 chemical entities had been isolated from 8 known and 18 unidentified *Amycolatopsis* species. Only 8 compounds, from the pargamicins and valgamicins families, possessed a simple cyclic peptide structure [4–6]. Since 2020, another 2 cyclic peptides have been identified, amycolatomycins A and B [7].

**Fig. 1:**
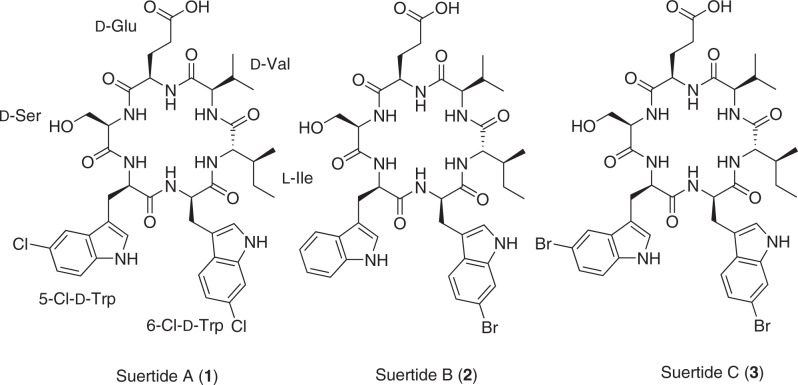
The molecular structures of suertides A–C (**1**–**3**) isolated from *Amycolatopsis* sp. MST-135876v3

*Amycolatopsis* sp. MST-135876 was isolated from soil collected from the riverbank of El Pont de Suert, 230 km east of Pamplona, Spain. Preliminary HPLC of a crude extract of the strain identified a metabolite profile unreported in *Amycolatopsis*. On passage, the strain lost its vitality and productivity, which were restored on mono-spore selection. The variant 3 subculture of the original isolate yielded a stable strain with high secondary metabolite productivity. Analysis of the metabolite profile revealed several compounds with UV–vis spectra commonly associated with the amino acid tryptophan (λ_max_ 192, 228, 284 nm) in addition to several others with UV–vis spectra comparable to the previously reported amycolatopsins [8]. This report describes the metabolic restoration and cultivation of MST-135876, and the isolation, spectroscopic characterisation, and biological profiling of three new cyclic hexapeptides, suertides A–C (**1**–**3**) (Fig. 1).

## Experimental section

### Mono-spore culture preparation

Culture MST-135876 was used to inoculate one ISP2 agar plate, which was then incubated in a dark temperature-controlled room at 28 °C. On day 3, individual colonies (10) were picked from the plate using a sterile toothpick, and individually streaked onto separate ISP2 daughter plates. The daughter plates were incubated at 28 °C for 7 days, at which point they were subsampled (1 g) and extracted in MeOH (2 ml) for 1 h and analysed by HPLC to determine their metabolite profiles. The residual culture from each daughter plate was preserved at −80 °C under glycerol (40%).

### Cultivation and extraction (1)

MST-135876v3 was cultured on ISP2 agar plates for 7 days at 28 °C. A spore suspension (H_2_O; 100 ml) was used to inoculate 260 × Modified Bennett’s (MS) agar plates (2.1 kg), which were incubated for 10 days at 28 °C, before being bagged and frozen at −20 °C for three days. The frozen plates were thawed at 28 °C for 14 h over a sieve, resulting in an aqueous extract (2000 ml) and dehydrated agar plates. The dehydrated agar plates were extracted in 3:1 CHCl_3_/MeOH (1 × 2300 ml; 1 × 2000 ml) and acetone (1 × 1800 ml). The organic solvent extractions were filtered, combined and reduced in vacuo to an aqueous slurry (400 ml) before being partitioned against ethyl acetate (2 × 1000 ml). After in vacuo reduction, the crude ethyl acetate extract (4.5 g) was dissolved in CHCl_3_ (150 ml), applied to a silica gel column (80 g) and eluted with a stepwise gradient of 0–100% MeOH in CHCl_3_, collecting a total of 16 fractions (500 ml). The compounds characterised by tryptophan-like UV spectra eluted in fractions A13 − A15. Fraction A15 (507.7 mg) was dissolved in MeOH (4 ml) and purified using preparative HPLC (Zorbax-SB C_18_, 250 × 21.5 mm, isocratic 36.25% MeCN, 36.25% MeOH, and 27.5% H_2_O containing 0.1% TFA, 20 ml min^−1^) to yield **1** (*t*_*R*_ = 8.02 min, 3.1 mg).

### Cultivation and extraction (2 and 3)

MST-135876v3 was cultured on ISP2 agar plates for 7 days at 28 °C. A spore suspension (H_2_O; 100 ml) was used to inoculate 250 × MS + 2% KBr agar plates (2.2 kg), which were incubated for 10 days at 28 °C, before being bagged and frozen at −20 °C for 2 days. The frozen plates were thawed at 28 °C for 24 h over a sieve, resulting in an aqueous extract (1800 ml) and dehydrated agar plates. The agar plates were extracted in 3:1 CHCl_3_/MeOH (3 × 2000 ml) and the organic solvent extractions were filtered, combined and reduced in vacuo to an aqueous slurry (400 ml) before being partitioned against ethyl acetate (2 × 1000 ml). The peptides were observed in both the aqueous and organic phases of the separation. The ethyl acetate extract was reduced in vacuo, dissolved in MeOH (150 ml) and partitioned against hexanes (2 × 300 ml). The MeOH fraction was reduced in vacuo to a residue. The MeOH from the hexanes-methanol partition and the aqueous fraction of the ethyl acetate-water partition were dissolved in 3:1 CHCl_3_ and MeOH (300 ml), applied to a silica gel column (80 g) and eluted with a stepwise gradient of 0–100% MeOH in CHCl_3_, collecting a total of 12 fractions (500 ml). The UV–vis spectra of the peptides were observed in fractions C10 and C11. Fraction C11 was dissolved in MeOH (4 ml) and fractionated by preparative HPLC (C_18_-enhanced polar selectivity, 250 × 22 mm, isocratic 36.25% MeCN, 36.25% MeOH, and 27.5% H_2_O containing 0.1% TFA, 20 ml min^−1^) to yield **2** (*t*_*R*_ = 7.65 min, 4.0 mg) and **3** (*t*_*R*_ = 13.63 min, 8.9 mg).

### Marfey’s analysis

To determine the absolute configuration of the amino acids, compound **1** (0.50 mg) was dissolved in H_2_O (100 μl) and HCl (5 M; 50 µl) was added. The solution was heated at 60 °C for 24 h, then dried under nitrogen prior to the addition of NaHCO_3_ solution (10 M; 50 µl) and Marfey’s reagent in acetone (1% *w/v*; 20 μl). The reaction mixture was heated at 60 °C for 2 h and the reaction neutralised with HCl (10 M; 25 µl) before analysis on LC-MS. Marfey’s conjugate standards for d- and l-valine, isoleucine, *allo*-isoleucine, serine, tryptophan, 5-chlorotryptophan, 6-chlorotryptophan, and glutamic acid were prepared and compared with the amino acids hydrolysed from **1**.

### Amino acid sequence determination

LC-MS was used to support the structure of **1** as determined by NMR. The peptide was first linearised according to a previous method, slightly modified [9]. Briefly, an aliquot (20 µl) of a solution of the peptide in MeOH (250 µg ml^−1^) was diluted to 100 µl with water and HCl (1.2 M; 100 µl) was added. The resulting solution was heated at 90 °C for 40 min to partially hydrolyse the peptide. The reaction was cooled on ice and quenched with an equimolar quantity of NaOH. The sample was dried in a vacuum centrifuge (Labconco) and redissolved in an aqueous solution of 5% MeCN / 0.1% formic acid (20 µl). LC-MS analysis was conducted on a Q Exactive Focus instrument (Thermo Fisher Scientific). Raw data returned from the laboratory were analysed with the software package Xcalibur Qual Browser 3.0.63 (Thermo Fisher Scientific) and manually sequenced de novo (Supplementary Fig. S27). The Orbitrap LC-MS data were acquired by the Thermo Fisher Proof of Concept Laboratory at Edith Cowan University, Perth, Australia.

## Results and discussion

The secondary metabolite distribution of MST-135876 was highly media-dependent, with **1** produced on only one of fifteen media, MS liquid medium. During these experiments, it was noticed that the vitality and metabolic productivity of the strain were unstable and diminished with iterative passage. A limited dilution spread of the original strain on agar identified 80% of the strains as non-producers and the productivity was stabilised by selection of a stable mono-spore, variant 3 (v3). MST-135876v3 was used in all subsequent cultivations, with optimal production of **1** observed on day 7 of a culture on MS agar. Supplementing MS agar with 2% KBr suppressed the production of **1**, while triggering the production of two novel non-polar analogues. Both the MS and MS + 2% KBr cultures were processed separately and extracted with acetone, the crude extract then partitioned between ethyl acetate and water followed by removal of the fats by hexane/methanol partition, provided an enriched fraction that was further fractionated by reversed phase C_18_ preparative HPLC to yield pure **1**–**3** (Supplementary Fig. S1).

16S rRNA gene sequence analysis indicated that the strain MST-135876v3 has 99.57% similarity to *Amycolatopsis xuchangensis* str. CFH S0322 [10]. The MST-135876v3 16S data also showed strong similarity to *Amycolatopsis magusensis* str. KT2025 (98.72%) [11], *Amycolatopsis albispora* str. WP1 (97.60%) [12], *Amycolatopsis jiguanensis* str. CFHS01580 (97.04%) [10], and *Amycolatopsis xylanica* str. CPCC 202699 (96.74%) [13]. A total of ten type strain *Amycolatopsis* species showed over 96% similarity to the 16S data for MST-135876v3 (Supplementary Table S1), hence the microbial species was tentatively identified as an *Amycolatopsis* species. The 16S sequence was submitted to GenBank under accession number OK487575.

**Fig. 2:**
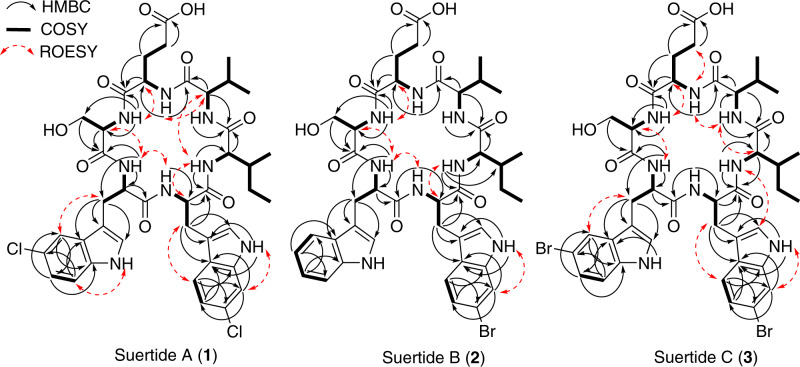
Key 2D NMR correlations for suertides A–C (**1**–**3**)

HRESI(+)MS analysis of **1** indicated a molecular formula C_41_H_50_Cl_2_N_8_O_9_ ([M + Na]^+^ *m/z* 891.2968, Δmmu −0.2). A distinctive isotopic pattern [M + H]^+^ *m/z* 869/871/873 with 9:6:1 relative intensities was observed (Supplementary Fig. S20), which is characteristic of dichlorinated compounds. The ^13^C NMR spectrum of **1** revealed 41 distinct peaks, while the ^1^H NMR peak integration suggested 50 protons, in agreement with the calculated chemical formula (Table 1, Supplementary Fig. S3). ^1^H – ^13^C HSQC, ^1^H – ^13^C HMBC, ^1^H – ^1^H COSY, and ^1^H – ^1^H ROESY NMR data were used to elucidate the molecular structure (Supplementary Figs. S4–S7). COSY correlations defined a spin system extending from Val-NH (*δ*_H_ 8.34) to Val-H-γ1/γ2 (*δ*_H_ 0.81/0.82), suggesting the presence of valine in the molecule (Fig. 2). An HMBC correlation from Val-H-α to *δ*_C_ 170.4 identified the Val carbonyl carbon. HMBC correlations from Ile-H-α (*δ*_H_ 4.00) to the Ile-carbonyl carbon (*δ*_C_ 172.2), Ile-C-β (*δ*_C_ 34.9), and Ile-C-γ1/γ2 (*δ*_C_ 25.0/14.6), and from Ile-H-*δ* (*δ*_H_ 0.73) to Ile-C-β and Ile-C-γ1, indicated the presence of isoleucine. COSY correlations from Ile-NH (*δ*_H_ 8.21) to Ile-H-α completed assignment of the amino acid. ROESY correlations between Ile-NH and Val-H-α suggested the two amino acids were adjacent. A second ROESY correlation between Ile-NH and 6-Cl-Trp-H-α (*δ*_H_ 4.72) was also observed. The 6-Cl-Trp-H-α proton showed HMBC correlations to the carbonyl carbon (*δ*_C_ 170.0), the β position (*δ*_C_ 28.6), and C-3 (*δ*_C_ 109.9). Further HMBC correlations were observed from 6-Cl-Trp-H-1 (*δ*_H_ 11.00) to C-2 (*δ*_C_ 124.6), C-3, C-3a (*δ*_C_ 126.5), and C-7a (*δ*_C_ 136.4). HMBC correlations from H-4 (*δ*_H_ 7.56) to C-7a and C-6 (*δ*_C_ 125.5), from H-5 (*δ*_H_ 6.98) to C-3a and C-7 (*δ*_C_ 110.7), and from H-7 (*δ*_H_ 7.33) to C-3a and C-5 (*δ*_C_ 118.5) suggested a 6-substituted tryptophan. This hypothesis was supported by strong reciprocal COSY correlations between H-4 and H-5. Accounting for the calculated formula that suggested 2 chlorine atoms, the amino acid was taken to be 6-Cl-Trp. The COSY and ROESY data from 6-Cl-Trp-NH (*δ*_H_ 7.37) to the H-α position were used to define the remaining constituents of the amino acid. The fourth amino acid in the sequence was identified from a diagnostic ROESY correlation between 5-Cl-Trp-NH (*δ*_H_ 8.66) and 6-Cl-Trp-NH. The 5-Cl-Trp-NH proton showed HMBC correlations to the C-α (*δ*_C_ 54.2) and C-β (*δ*_C_ 26.9) positions. COSY correlations were also observed between H-α (*δ*_H_ 4.26) and diastereotopic H-βa/b (*δ*_H_ 3.20/2.90). HMBC correlations from H-βa/b to C-2 (*δ*_C_ 125.3), C-3 (*δ*_C_ 110.6), and C-3a (*δ*_C_ 128.3), and from H-2 (*δ*_H_ 7.20) to C-3, C-3a and C-7a (*δ*_C_ 134.5), indicated the presence of a second tryptophan residue in the molecule. Diagnostic HMBC correlations from H-4 (*δ*_H_ 7.57) to C-6 (*δ*_C_ 120.8) and C-7a, in addition to the correlations from H-6 (*δ*_H_ 7.04) to C-4 (*δ*_C_ 117.4) and C-7a, and from H-7 (*δ*_H_ 7.33) to C-3a and non-protonated C-5 (*δ*_C_ 123.1) suggested that the second tryptophan was 5-chloro- substituted. This was further evidenced by the strong reciprocal correlations between H-6 and H-7 in the COSY spectrum. An HMBC correlation from H-α position to a carbonyl carbon (*δ*_C_ 170.8) completed assignment of the amino acid. A ROESY correlation between 5-Cl-Trp-NH and Ser-H-α (*δ*_H_ 4.12) was used as a starting point for the fifth amino acid. HMBC correlations from the Ser-H-α to the carbonyl carbon (*δ*_C_ 170.3) and Ser-C-β (*δ*_C_ 60.3) defined the extent of the amino acid. This was supported by COSY correlations between Ser-H-α and Ser-H-β (*δ*_H_ 3.27) and Ser-NH (*δ*_H_ 8.19). The ^1^H and ^13^C chemical shifts of the β position are typical of those found in serine, although the presence of an OH group was not observed in the ^1^H NMR spectrum of **1**. As such, this amino acid was tentatively characterised as serine. ROESY correlations from Ser-NH to Glu-H-α (*δ*_H_ 4.40), Glu-H-βb (*δ*_H_ 1.79), and Glu-H-γ (*δ*_H_ 2.18) were observed, indicating the start of the sixth amino acid. The connectivity of these residues was confirmed by HMBC correlations from Glu-H-α to a carbonyl carbon (*δ*_C_ 170.3), C-β (*δ*_C_ 28.5) and C-γ (*δ*_C_ 29.7). An HMBC correlation from Glu-H-γ to a second carbonyl carbon (*δ*_C_ 173.9) and the presence of a downfield chemical shift associated with an acidic hydroxy group (*δ*_H_ 12.10), confirmed the identity of the final amino acid as glutamic acid. A COSY correlation between Glu-H-α and Glu-NH (*δ*_H_ 7.28) was used to identify the final amide group. ROESY correlations between Glu-NH (*δ*_H_ 7.28) and Val-NH, Val-H-α, and Val-H-γ1/2 completed the cyclic hexapeptide core. De novo sequencing by LC-MS/MS confirmed the amino acid sequence determined from the NMR data (Supplementary Fig. S32). A Marfey’s analysis was undertaken to determine the absolute configurations of the amino acids present in **1** [14]. Using LC-MS to compare the retention times of the Marfey’s-conjugated amino acids liberated from acid-catalysed hydrolysis of **1** to those of amino acid standards (Supplementary Figs. S26−S31), we identified the presence of d-Ser, 5-Cl-d-Trp, 6-Cl-d-Trp, l-Ile, d-Val, and d-Glu. Taken together, the absolute configuration of **1** was confirmed, as depicted in Fig. 1.

**Table 1:** ^1^H (600 MHz) and ^13^C (150 MHz) NMR data for suertides A–C (**1**–**3**) in DMSO-*d*_6_

		Suertide A (1)		Suertide B (2)		Suertide C (3)	
Unit	Pos.	δ_C_	δ_H_, mult (*J* in Hz)	δ_C_	δ_H_, mult (*J* in Hz)	δ_C_	δ_H_, mult (*J* in Hz)
l-Ser	NH		8.19, d (6.1)		8.19, d (5.7)		8.20, m
	CO	170.3		170.3		170.4	
	α	56.2	4.12, q (6.5)	56.3	4.12, m	56.2	4.11, q
	β	60.3	3.27, m	60.4	3.28, m	60.3	3.26, m
	OH						
5-X-d-Trp	NH		8.66, d (8.2)		8.65, d (8.0)		8.66, d (8.1)
	CO	170.8		170.9		170.8	
	α	54.2	4.26, m	54.3	4.26, m	54.2	4.25, m
	βa	26.9	3.20, dd (15.2, 3.7)	27.0	3.25, m	26.8	3.21, dd (15.0, 2.9)
	βb		2.90, dd (15.2, 10.8)		2.89, dd (15.0, 11.0)		2.90, dd (15.0, 10.8)
	1		11.00, d (2.2)	10.8			11.00, d (1.9)
	2	125.3	7.20, d (2.2)	123.2	7.11^b^, m	125.1	7.19, d (2.3)
	3	110.6		110.6		110.5	
	3a	128.3		127.1		129.0	
	4	117.4	7.57^a^, m	118.0	7.53^c^, d (7.8)	120.4	7.71, d (1.9)
	5	123.1		118.3	6.98, m	111.0	
	6	120.8	7.04, dd (8.6, 2.0)	120.9	7.04, m	123.3	7.15, dd (8.6, 1.9)
	7	112.8	7.33^b^, m	111.3	7.31, d (8.1)	113.3	7.29, d (8.6)
	7a	134.5		136.0		134.7	
6-Y-d-Trp	NH		7.37, d (2.0)		7.37, d (7.1)		7.37, d (7.1)
	CO	170.0		170.0		170.0	
	α	53.5	4.72, m	53.4	4.73, m	53.5	4.73, m
	βa	28.6	3.07, dd (14.0, 8.6)	28.6	3.09, dd (14.1, 8.4)	28.4	3.07, dd (14.0, 8.6)
	βb		2.98, dd (14.0, 4.7)		2.99, dd (14.1, 4.9)		2.98, dd (14.0, 4.8)
	1		11.00, d (1.9)		10.97, d (2.1)		11.00, d (1.9)
	2	124.6	7.09, d (2.3)	124.5	7.09, m	124.6	7.08, d (2.3)
	3	109.9		109.9		109.9	
	3a	126.5		126.8		126.7	
	4	120.1	7.56^a^, m	120.5	7.52^c^, d (5.2)	120.5	7.51, d (8.4)
	5	118.5	6.98, dd (8.5, 1.9)	121.0	7.11^b^, m	121.0	7.10, dd (8.4, 1.7)
	6	125.5		113.6		113.6	
	7	110.7	7.33^b^, m	113.6	7.48, d (1.7)	113.5	7.48, d (1.7)
	7a	136.4		136.8		136.8	
l-Ile	NH		8.21, d (7.6)		8.21, d (7.3)		8.20, m
	CO	172.2		172.2		172.2	
	α	57.9	4.00, dd (8.8, 7.6)	57.9	4.00, dd (8.7, 7.6)	57.9	4.00, dd (8.7, 7.6)
	β	34.9	1.50, m	34.9	1.51, m	34.9	1.50, m
	γ1a	25.0	1.17, m	24.9	1.19, m	24.9	1.18, m
	γ1b		0.91, m		0.93, m		0.92, m
	γ2	14.6	0.47, d (6.8)	14.6	0.49, d (6.9)	14.6	0.47, d (6.8)
	δ	10.7	0.73, t (7.4)	10.7	0.75, t (7.4)	10.7	0.73, t (7.4)
d-Val	NH		8.34, d (8.8)		8.34, d (8.8)		8.35, d (8.8)
	CO	170.4		170.4		170.3	
	α	58.0	4.07, dd (8.8, 4.5)	58.0	4.07, dd (8.8, 4.5)	58.0	4.07, dd (8.8, 4.5)
	β	29.0	2.26, m	29.0	2.26, m	28.6	2.26, m
	γ1	19.4	0.81, d (6.9)	19.4	0.82^a^, d (2.9)	19.4	0.81^a^, d (2.9)
	γ2	17.0	0.82, d (6.9)	16.9	0.82^a^, d (2.9)	16.9	0.83^a^, d (2.9)
d-Glu	NH		7.28, m (7.5)		7.27, d (7.8)		7.27, d (7.6)
	CO	170.3		170.3		170.3	
	α	51.0	4.40, q (6.7)	51.0	4.40, q (6.7)	51.0	4.41, d (6.7)
	βa	28.5	1.89, m	28.4	1.89, m	29.0	1.88, m
	βb		1.79, m		1.78, m		
	γa	29.7	2.18, t (8.3)	29.7	2.18, t (8.3)	29.7	1.80, m
	γb						2.18, t (8.3)
	δ	173.9		173.9		173.9	
	OH		12.10, br s		12.10, br s		12.09, br s

^a–c^Signals overlapping

HRESI(+)MS analysis of **2** revealed a molecular formula C_41_H_51_BrN_8_O_9_ ([M + Na]^+^ *m/z* 901.2855, Δmmu 0.0). A distinctive isotopic pattern *m/z* 901/903 with 1:1 relative intensity (Supplementary Fig. S21), suggested the incorporation of one bromine atom into the cyclic peptide. The NMR data for **2** were very similar to those for **1**, with the only significant difference being the presence of an additional aromatic methine proton (H-5; *δ*_H_ 6.98) on the tryptophan residue adjacent to serine, suggesting the presence of non-halogenated tryptophan. This was supported by COSY correlations between H-4 (*δ*_H_ 7.53) and H-5, between H-5 and H-6 (*δ*_H_ 7.04), and between H-6 and H-7 (*δ*_H_ 7.31) (Fig. 2). As the remaining inter- and intra-amino acid correlations were consistent with the previously described non-substituted tryptophan amino acids, this indicated that the compound was a monobromo-derivative of **1**. A chemical shift comparison between the 6-substituted tryptophan residues of **1** and **2** revealed an upfield shift in C-6 from *δ*_C_ 125.5 (**1**) to *δ*_C_ 113.6 (**2**), consistent with the additional shielding provided by bromine compared to chlorine. A downfield shift was observed for the atoms *ortho* to the halogenation site, with position 5 changing from *δ*_H_ 6.98/*δ*_C_ 118.5 (**1**) to *δ*_H_ 7.11/*δ*_C_ 121.0 (**2**), at position 7, the chemical shifts changed from *δ*_H_ 7.33/*δ*_C_ 110.7 (**1**) to *δ*_H_ 7.48/*δ*_C_ 113.8 (**2**), and there was no notable change at the 4-position. An HMBC correlation from H-5 (*δ*_H_ 7.11) to C-7, as well as the reciprocal COSY correlations between H-4 and H-5, indicated that this amino acid was 6-Br-Trp. Given their close biosynthetic relationship, that the absolute configuration of **2** was tentatively assigned to be the same as **1**. Taken together, the HRESI(+)MS and NMR data confirmed the structure of **2** to be *cyclo*(d-Ser, d-Trp, 6-Br-d-Trp, l-Ile, d-Val, d-Glu), as shown in Fig. 1.

HRESI(−)MS analysis of **3** suggested a molecular formula C_41_H_50_Br_2_N_8_O_9_ ([M − H]^−^ *m/z* 955.1995, Δmmu 0.0). The spectrum contained a distinctive isotopic pattern *m/z* 955/957/959 with the relative intensity of 1:2:1 (Supplementary Fig. S22), indicative of a dibrominated compound. A comparison of the 1D and 2D NMR data obtained for **3** with those obtained for **1** revealed that the compounds were nearly identical. In the 6-substituted tryptophan, there was a characteristic upfield shift of C-6 from *δ*_C_ 125.5 (**1**) to *δ*_C_ 113.6 (**3**), suggesting substitution by bromine rather than chlorine. A downfield shift of the atoms *ortho* to the bromine-substituted carbon was observed, with position 5 changing from *δ*_H_ 6.98 / *δ*_C_ 118.5 (**1**) to *δ*_H_ 7.10 / *δ*_C_ 121.0 (**3**), and position 7 changing from *δ*_H_ 7.33 / *δ*_C_ 110.7 (**1**) to *δ*_H_ 7.48 / *δ*_C_ 113.5 (**3**). The key correlations from H-5 to C-3a (*δ*_C_ 126.7) and C-7 were observed, as were reciprocal COSY correlations between H-4 (*δ*_H_ 7.51) and H-5. Similarly, in the 5-substituted tryptophan, there was a change from *δ*_C_ 123.1 (**1**) to 111.0 (**3**) at the 5-position. Downfield shifts were also noted in the positions *ortho* to the brominated carbon, with C-4 changing from *δ*_H_ 7.57 / *δ*_C_ 117.4 (**1**) to *δ*_H_ 7.71 / *δ*_C_ 120.4 (**3**), and C-6 changing from *δ*_H_ 7.04 / *δ*_C_ 120.8 (**1**) to *δ*_H_ 7.15 / *δ*_C_ 123.3 (**3**). The characteristic HMBC correlations from H-6 to C-4 and C-7a (*δ*_C_ 134.7), in addition to the COSY correlations between H-6 and H-7 (*δ*_H_ 7.29), were used to confirm the amino acid was 5-bromo-substituted. Taken together, the HRESI(−)MS and NMR data confirmed that the structure of **3** was *cyclo*(d-Ser, 5-Br-d-Trp, 6-Br-d-Trp, l-Ile, d-Val, d-Glu), as shown in Fig. 1.

### Biological activity

The suertides were evaluated for in vitro biological activity in antibacterial, antifungal, antiprotozoal, herbicidal, and antitumour bioassays and found to be selective antibacterial compounds. All compounds showed strong biological activity against *Bacillus subtilis* (ATCC 6633) (MIC 1.6 μg ml^−1^, Table 2), while differences in antibacterial activity were observed against *Staphylococcus aureus* (ATCC 25923), with the presence of brominated tryptophan residues resulting in reduced activity (3.1, 6.3, and 25.0 μg ml^−1^, for **1**, **2**, and **3**, respectively). The compounds were also tested against methicillin-resistant *S. aureus* (MRSA, ATCC 33592), revealing increased potency of **1** (1.6 μg ml^−1^), comparable activity for **2** (6.3 μg ml^−1^) and no activity for **3**. The reported compounds showed no activity up to 100 µg ml^−1^ against the Gram-negative bacterium *Escherichia coli* (ATCC 25922), the fungus *Candida albicans* (ATCC 10231), a mouse myeloma cell line (NS-1), a human fibroblast cell line (NFF), the protozoan *Tritrichomonas foetus* (strain KV-1) or the monocotyledonous plant *Eragrostis tef* (teff).

**Table 2:** In vitro bioassay data for compounds **1**–**3**

Compound	MIC (μg ml^−1^)		
	BS^a,^	SA^b^	MRSA^c^
**1**	1.6	3.1	1.6
**2**	1.6	6.3	6.3
**3**	1.6	25	>100

Experiments were conducted in triplicate to determine MIC, MIC was taken at 48 h

^a^*Bacillus subtilis* (ATCC 6633)

^b^*Staphylococcus aureus* (ATCC 25923)

^c^Methicillin-resistant *Staphylococcus aureus* (ATCC 33592)

Actinobacteria-derived antibacterial chlorinated peptides are a large group of small molecules that continues to grow. However, until recently, few compounds were associated with the genus *Amycolatopsis* [3]. The suertides are a family of antibacterial cyclic hexapeptides that contain more than two-thirds of the constitutive amino acids in the rarer non-proteogenic d-configuration and represent the second example in of two adjacent Trp moieties within a cyclic peptide from *Amycolatopsis*. The amycolatomycins, recently isolated by the Stadler laboratory, contain the same core amino acid units as suertide A, but with differing primary sequence, stereochemical configuration, and the presence of a distinct 2,6-dichloro-l-Trp residue [7]. To date, all the non-thiazolyl cyclic peptides isolated from *Amycolatopsis* have shown varying degrees of activity against MRSA [4–6] except for amycolatomycin A, which showed only weak antibacterial activity against *B. subtilis* (33.4 μg ml^−1^) [7]. More distantly related ditryptophan-containing metabolites include the cyclic heptapeptide argyrin A, from the myxobacterium *Archangium gephyra* [15], the chlorotryptophan-containing cyclic heptadepsipeptide krisynomycin, from *Streptomyces canus* [16], the cyclic nonapeptide propeptin, from *Microbispora* sp. [17], and the cyclic octadepsipeptides telomycins, which contain adjacent tryptophanyl and dihydrotryptophanyl moieties within the macrocycle [18]. Alone among these cyclic peptides, the suertides are the sole examples featuring two adjacent d-Trp moieties.

In conclusion, three new halogenated cyclo-hexapeptides were isolated from a putative *Amycolatopsis* sp. collected from a riverbank in Spain. All three compounds display antibacterial activity, with two displaying strong activity against MRSA and no cytotoxicity against mammalian cell lines up to 100 µg ml^−1^. Ultimately, this study demonstrated the ongoing utility of novel, soil derived actinobacteria in the quest for chemical novelty in drug discovery.

### Physical characterisation

Suertide A (**1**): white powder; $[a]\frac{{20}}{D}$  + 25 (*c* 0.03, MeOH); UV (MeCN) λ_max_ (log ε) 200 (5.06); 230 (4.86); 288 (4.02) nm; HRMS *m/z* 891.2968; calcd. for C_41_H_50_Cl_2_N_8_O_9_Na^+^ [M + Na]^+^, 891.2968.

Suertide B (**2**): white powder; $[a]\frac{{20}}{D}$  + 9 (*c* 0.02, MeOH); UV (MeCN) λ_max_ (log ε) 200 (5.06); 230 (4.86); 288 (4.02) nm; IR (ATR) ν_max_ 3271, 2961, 2922, 2359, 1643, 1542, 1456, 744, 660 cm^–1^; HRMS *m/z* 901.2855; calcd. for C_41_H_51_BrN_8_O_9_Na^+^ [M + Na]^+^, 901.2855.

Suertide C (**3**): white powder; $[a]\frac{{20}}{D}$  + 23 (*c* 0.09, MeOH); UV (MeCN) λ_max_ (log ε) 200 (5.06); 230 (4.86); 288 (4.02) nm; IR (ATR) ν_max_ 3285, 2961, 2925, 2358, 1626, 1536, 1225, 795, 686 cm^–1^; HRMS *m/z* 955.1995; calcd. for C_41_H_49_Br_2_N_8_O_9_^−^ [M − H]^−^, 955.1995.

## Supplementary information

Supplementary Information

## References

[1] Lechevalier M, Prauser H, Labeda D, Ruan J-S (1986). Two new genera of nocardioform actinomycetes: *Amycolata* gen. nov. and *Amycolatopsis* gen. nov. Int J Syst Evol Micr.

[2] List of prokaryotic names with standing in nomenclature, vol. 2021. Leibniz Institute: Leibniz, Germany, 1998, https://lpsn.dsmz.de/.

[3] Song Z, Xu T, Wang J, Hou Y, Liu C, Liu S (2021). Secondary metabolites of the genus *Amycolatopsis*: structures, bioactivities and biosynthesis. Molecules..

[4] Hashizume H, Iijima K, Yamashita K, Kimura T, Wada S-I, Sawa R (2018). Valgamicin C, a novel cyclic depsipeptide containing the unusual amino acid cleonine, and related valgamicins A, T and V produced by *Amycolatopsis* sp. ML1-hF4. J Antibiot.

[5] Hashizume H, Sawa R, Yamashita K, Nishimura Y, Igarashi M (2017). Structure and antibacterial activities of new cyclic peptide antibiotics, pargamicins B, C and D, from *Amycolatopsis* sp. ML1-hF4. J Antibiot.

[6] Igarashi M, Sawa R, Kinoshita N, Hashizume H, Nakagawa N, Homma Y (2008). Pargamicin a, a novel cyclic peptide antibiotic from *Amycolatopsis* sp. J Antibiot.

[7] Primahana G, Risdian C, Mozef T, Wink J, Surup F, Stadler M (2021). Amycolatomycins A and B, cyclic hexapeptides isolated from an *Amycolatopsis* sp. 195334CR. Antibiotics.

[8] Khalil ZG, Salim AA, Vuong D, Crombie A, Lacey E, Blumenthal A (2017). Amycolatopsins A-C: antimycobacterial glycosylated polyketide macrolides from the Australian soil *Amycolatopsis* sp. MST-108494. J Antibiot.

[9] Fisher MF, Mylne JS (2019). Sequencing orbitides by acid-mediated ring cleavage followed by tandem mass spectrometry. J Proteome Res.

[10] Huang J-R, Ming H, Li S, Zhao Z-L, Meng X-L, Zhang J-X (2016). *Amycolatopsis xuchangensis* sp. nov. and *Amycolatopsis jiguanensis* sp. nov., isolated from soil. A Van Leeuw J Micro.

[11] Camas M, Sahin N, Sazak A, Spröer C, Klenk H-P (2013). *Amycolatopsis magusensis* sp. nov., isolated from soil. Int J Syst Evol Micr.

[12] Zhang G, Wang L, Li J, Zhou Y (2016). *Amycolatopsis albispora* sp. nov., isolated from deep-sea sediment. Int J Sys Evol Micr.

[13] Chen J, Su J-J, Wei Y-Z, Li Q-P, Yu L-Y, Liu H-Y (2010). *Amycolatopsis xylanica* sp. nov., isolated from soil. Int J Sys Evol Micr.

[14] Marfey P, Ottesen M (1984). Determination of D-amino acids. I. Hydrolysis of DNP-L-amino acid methyl esters with carboxypeptidase-Y. Carlsberg Res Commun.

[15] Sasse F, Steinmetz H, Schupp T, Petersen F, Memmert K, Hofmann H (2002). Argyrins, immunosuppressive cyclic peptides from myxobacteria I. production, isolation, physico-chemical and biological properties. J Antibiot.

[16] Pérez-Bonilla M, Oves-Costales D, González I, de la Cruz M, Martín JS, Vicente F (2020). Krisynomycins, imipenem potentiators against methicillin-resistant *Staphylococcus aureus*, produced by *Streptomyces canus*. J Nat Prod.

[17] Esumi Y, Suzuki Y, Itoh Y, Uramoto M, Kimura K-I, Goto M (2002). Propeptin, a new inhibitor of prolyl endopeptidase produced by microbispora II. Determination of chemical structure. J Antibiot.

[18] Sheehan JC, Mania D, Nakamura S, Stock JA, Maeda K (1968). The structure of telomycin. J Am Chem Soc.

