# Supplementary material for: Suertides A–C: selective antibacterial cyclic hexapeptides from Amycolatopsis sp. MST-135876v3
Source: J Antibiot (Tokyo). 2022 Jul 26;75(9):483–90. doi: 10.1038/s41429-022-00544-4 (PMC9359914; doi:10.1038/s41429-022-00544-4)
Supplement: Supplementary file 1 — Supplementary Information [file 41429_2022_544_MOESM1_ESM.docx]

**SUPPORTING INFORMATION**

**Suertides A – C: selective antibacterial cyclic hexapeptides from *Amycolatopsis* sp. MST-135876v3**

Heather J. Lacey^1,2*^, Rachel Chen^1^, Daniel Vuong^1^, Mark F. Fisher^3^, Ernest Lacey^1,4^, Peter Rutledge^2^
and Andrew M. Piggott^4^

*^1^ Microbial Screening Technologies, Smithfield, NSW 2164, Australia*

*^2^ School of Chemistry, The University of Sydney, NSW 2006, Australia*

*^3^ School of Molecular and Life Sciences, Curtin University, WA 6102, Australia*

*^4^ School of Natural Sciences, Macquarie University, NSW 2109, Australia*

# Figures

[**Figure S1.** The extraction and purification sequence for MST-135876v3. 5](#_Toc106707506)

[**Figure S2.** Phylogenetic relationships of *Amycolatopsis* sp. MST-135876v3. The phylogenetic tree was generated using NCBI Tree View. 7](#_Toc106707507)

[**Figure S3.** ^1^H NMR spectrum (600 MHz, DMSO-*d*_6_) of suertide A (**1**). 14](#_Toc106707508)

[**Figure S4.** ^13^C NMR spectrum (150 MHz, DMSO-*d*_6_) of suertide A (**1**). 15](#_Toc106707509)

[**Figure S5**. HSQC NMR spectrum (600 MHz, DMSO-*d*_6_) of suertide A (**1**). 16](#_Toc106707510)

[**Figure S6.** HMBC NMR spectrum (600 MHz, DMSO-*d*_6_) of suertide A (**1**). 17](#_Toc106707511)

[**Figure S7.** COSY NMR spectrum (600 MHz, DMSO-*d*_6_) of suertide A (**1**). 18](#_Toc106707512)

[**Figure S8.** ROESY NMR spectrum (600 MHz, DMSO-*d*_6_) of suertide A (**1**). 19](#_Toc106707513)

[**Figure S9**. ^1^H NMR spectrum (600 MHz, DMSO-*d*_6_) of suertide B (**2**). 20](#_Toc106707514)

[**Figure S10.** ^13^C NMR spectrum (150 MHz, DMSO-*d*_6_) of suertide B (**2**). 21](#_Toc106707515)

[**Figure S11.** HSQC NMR spectrum (600 MHz, DMSO-*d*_6_) of suertide B (**2**). 22](#_Toc106707516)

[**Figure S12.** HMBC NMR spectrum (600 MHz, DMSO-*d*_6_) of suertide B (**2**). 23](#_Toc106707517)

[**Figure S13.** COSY NMR spectrum (600 MHz, DMSO-*d*_6_) of suertide B (**2**). 24](#_Toc106707518)

[**Figure S14.** ROESY NMR spectrum (600 MHz, DMSO-*d*_6_) of suertide B (**2**). 25](#_Toc106707519)

[**Figure S15**. ^1^H NMR spectrum (600 MHz, DMSO-*d*_6_) of suertide C (**3**). 26](#_Toc106707520)

[**Figure S16.** ^13^C NMR spectrum (150 MHz, DMSO-*d*_6_) of suertide C (**3**). 27](#_Toc106707521)

[**Figure S17.** HSQC NMR spectrum (600 MHz, DMSO-*d*_6_) of suertide C (**3**). 28](#_Toc106707522)

[**Figure S18.** HMBC NMR spectrum (150 MHz, DMSO-*d*_6_) of suertide C (**3**). 29](#_Toc106707523)

[**Figure S19.** COSY NMR spectrum (600 MHz, DMSO-*d*_6_) of suertide C (**3**). 30](#_Toc106707524)

[**Figure S20.** ROESY NMR spectrum (600 MHz, DMSO-*d*_6_) of suertide C (**3**). 31](#_Toc106707525)

[**Figure S21**. HRMS spectra of suertide A (**1**). Left: [M + Na]^+^. Right: [M + H]^+^. 32](#_Toc106707526)

[**Figure S22.** HRMS spectrum of suertide B (**2**). 33](#_Toc106707527)

[**Figure S23.** HRMS spectrum of suertide C (**3**). 34](#_Toc106707528)

[**Figure S24.** UV-vis spectrum of suertide A (**1**) in MeCN. 35](#_Toc106707529)

[**Figure S25.** UV-vis spectrum of suertide B (**2**) in MeCN. 35](#_Toc106707530)

[**Figure S26**. UV-vis spectrum of suertide C (**3**) in MeCN. 35](#_Toc106707531)

[**Figure S27.** HPLC traces (340 nm) of hydrolysed suertide A (**1**) (top) and l- and d-serine (bottom) after derivatisation with Marfey’s reagent 37](#_Toc106707532)

[**Figure S28.** HPLC traces (340 nm) of hydrolysed suertide A (**1**) (top) and l- and d-, 5-Cl and 6-Cl-tryptophan (bottom) after derivatisation with Marfey’s reagent. 38](#_Toc106707533)

[**Figure S29.** HPLC traces (340 nm) of hydrolysed suertide A (**1**) (top) and l- and d-isoleucine (bottom) after derivatisation with Marfey’s reagent. 39](#_Toc106707534)

[**Figure S30.** HPLC traces (340 nm) of hydrolysed suertide A (**1**) (top), l-*allo*-isoleucine (middle) and l-isoleucine (bottom) after derivatisation with Marfey’s reagent. 40](#_Toc106707535)

[**Figure S31.** HPLC traces (340 nm) of hydrolysed suertide A (**1**) (top) and l- and d-valine (bottom) after derivatisation with Marfey’s reagent. 41](#_Toc106707536)

[**Figure S32.** HPLC traces (340 nm) of hydrolysed suertide A (**1**) (top) and l-, d-glutamic acid (bottom) after derivatisation with Marfey’s reagent. 42](#_Toc106707537)

[**Figure S33.** LC-MS data for suertide A (**1**) after linearisation with HCl. 43](#_Toc106707538)

[**Figure S34.** Infrared spectrum of suertide B (**2**). 43](#_Toc106707539)

[**Figure S35.** Infrared spectrum of suertide C (**3**). 44](#_Toc106707540)

# Tables

[**Table S1.** Most similar species to MST-135876v3 based on a BLAST search of the 16S rRNA type strain gene sequences in the Combined 16S Database. 6](#_Toc106707541)

[**Table S2.** ^1^H (600 MHz), ^13^C (150 MHz), HMBC, COSY, and ROESY NMR data for suertide A (**1**) in DMSO-*d_6_*. 8](#_Toc106707542)

[**Table S3.** ^1^H (600 MHz), ^13^C (150 MHz), HMBC, COSY, and ROESY NMR data for suertide B (**2**) in DMSO-*d_6_*. 10](#_Toc106707543)

[**Table S4.** ^1^H (600 MHz), ^13^C (150 MHz), HMBC, COSY, and ROESY NMR data for suertide C (**3**) in DMSO-*d_6_*. 12](#_Toc106707544)

[**Table S5.** The retention times and ions mass used to determine the absolute configuration of suertide A (**1**). 36](#_Toc106707545)

## General Experimental

NMR spectra were acquired in DMSO-*d*_6_ on a Bruker Avance II DRX-600K 600 MHz spectrometer at 25 °C. NMR spectra were processed using Topspin 4.1 and referenced to the residual solvent signals (DMSO-*d*_6_; δ_H_ 2.49/δ_C_ 39.5). High-resolution electrospray ionisation mass spectra (HRESIMS) were acquired by direct infusion in MeCN on either a Bruker Apex Qe 7T Fourier Transform Ion Cyclotron Resonance mass spectrometer equipped with an Apollo II ESI/MALDI dual source or a Q Exactive Plus hybrid quadrupole-Orbitrap mass spectrometer. Electrospray ionisation (ESI) mass spectrometry was carried out with an Agilent 1260 Infinity HPLC series equipped with an Agilent 6120 Infinity series mass detector in both positive and negative ion modes. All HPLC and LC-MS data were analysed with in-house software COMET [139], respectively. Chiroptical measurements were acquired in MeOH with a Perkin-Elmer Model 341 polarimeter in a 50 × 5 mm cell. UV-vis spectra were acquired in MeCN on a Varian Cary 4000 spectrophotometer between 200 – 600 nm with a cell size of 10 × 10 mm. FT-IR spectra were obtained with Jasco FT/IR-4700 type A and processed with Spectra Manager™ Suite software.

Analytical HPLC was performed on a gradient Agilent 1260 Infinity quaternary HPLC system. The column was an Agilent Zorbax SB-C18 (2.1 × 50 mm, 1.8 µm) eluted with a 0.6 ml min^−1^ gradient of 10−100% MeCN/H_2_O (0.01% TFA) over 8.33 min. Preparative HPLC was performed on a gradient Shimadzu HPLC system comprising of two LC-8 preparative liquid pumps with a static mixer, SPD-M10AVP diode array detector and SCL-10AVP system controller with standard Rheodyne injection port. The columns used in the purification of the metabolites were selected from either a Vydac C_18_ column (50 × 100 mm, 5 µm; Grace Discovery), a Zorbax SB-C18 column (50 × 150 mm, 5 µm; Agilent) or an Alltima C_18_ (22 × 250 mm, 5 µm, Grace Discovery) isocratically with MeCN/H_2_O mixtures containing 0.01% TFA modifier.

Analytical LCMS was performed on an Agilent 1260 Infinity series HPLC equipped with an Agilent 6130B single quadrupole mass detector in both positive and negative ion modes. The column was an Agilent Zorbax C_18_ (2.1 × 50 mm, 1.8 µm) eluted with a 0.5 ml min^−1^ gradient of 10−100% MeCN/H_2_O (0.01% TFA) over 10 min.

## KBr Feeding Study

Ten MS agar plates containing either 0%, 0.1%, 0.5%, 1%, and 2% *w/v* of KBr were prepared and inoculated with 1 ml of spore suspension prior to incubation in a temperature-controlled room (28 ℃) for 7 days. On day 10, subsamples were taken from each plate and extracted with MeOH (2 ml) over 3 h. The subsamples were analysed by LC-MS (Zorbax C_18_, gradient 10% − 100% H_2_O/MeCN containing 0.01% formic acid, 0.5 ml min^−1^). Further UV-vis spectrum analysis of the HPLC data was conducted using COMET software [1].

**Figure S1.** The extraction and purification sequence for MST-135876v3.

**Table S1.** Most similar species to MST-135876v3 based on a BLAST search of the 16S rRNA type strain gene sequences in the Combined 16S Database.

| **Description** | **Scientific Name** | **Max Score** | **Total Score** | **Query Cover** | **E value** | **Per. ident** | **Acc. Len** | **Accession** |
| --- | --- | --- | --- | --- | --- | --- | --- | --- |
| *Amycolatopsis xuchangensis* strain CFH S0322 16S ribosomal RNA, partial sequence | *Amycolatopsis xuchangensis* | 1286 | 1286 | 100% | 0 | 99.57 | 1517 | [NR_164869.1](https://www.ncbi.nlm.nih.gov/nucleotide/NR_164869.1?report=genbank&log$=nucltop&blast_rank=1&RID=ASCN166V013) |
| *Amycolatopsis magusensis* strain KT2025 16S ribosomal RNA, partial sequence | *Amycolatopsis magusensis* | 1253 | 1253 | 100% | 0 | 98.72 | 1471 | [NR_109060.1](https://www.ncbi.nlm.nih.gov/nucleotide/NR_109060.1?report=genbank&log$=nucltop&blast_rank=2&RID=ASCN166V013) |
| *Amycolatopsis albispora* strain WP1 16S ribosomal RNA, partial sequence | *Amycolatopsis albispora* | 1210 | 1210 | 100% | 0 | 97.6 | 1481 | [NR_152021.1](https://www.ncbi.nlm.nih.gov/nucleotide/NR_152021.1?report=genbank&log$=nucltop&blast_rank=3&RID=ASCN166V013) |
| *Amycolatopsis jiguanensis* strain CFHS01580 16S ribosomal RNA, partial sequence | *Amycolatopsis jiguanensis* | 1188 | 1188 | 100% | 0 | 97.04 | 1475 | [NR_164870.1](https://www.ncbi.nlm.nih.gov/nucleotide/NR_164870.1?report=genbank&log$=nucltop&blast_rank=4&RID=ASCN166V013) |
| *Amycolatopsis xylanica* strain CPCC 202699 16S ribosomal RNA, partial sequence | *Amycolatopsis xylanica* | 1171 | 1171 | 100% | 0 | 96.74 | 1451 | [NR_116718.1](https://www.ncbi.nlm.nih.gov/nucleotide/NR_116718.1?report=genbank&log$=nucltop&blast_rank=5&RID=ASCN166V013) |
| *Amycolatopsis marina* strain Ms392A 16S ribosomal RNA, partial sequence | *Amycolatopsis marina* | 1168 | 1168 | 100% | 0 | 96.47 | 1485 | [NR_044465.1](https://www.ncbi.nlm.nih.gov/nucleotide/NR_044465.1?report=genbank&log$=nucltop&blast_rank=6&RID=ASCN166V013) |
| *Amycolatopsis nigrescens* CSC17Ta-90 16S ribosomal RNA, partial sequence | *Amycolatopsis nigrescens* CSC17Ta-90 | 1158 | 1158 | 100% | 0 | 96.32 | 1409 | [NR_043880.1](https://www.ncbi.nlm.nih.gov/nucleotide/NR_043880.1?report=genbank&log$=nucltop&blast_rank=7&RID=ASCN166V013) |
| *Amycolatopsis palatopharyngis* strain 1Bdz 16S ribosomal RNA, partial sequence | *Amycolatopsis palatopharyngis* | 1157 | 1157 | 100% | 0 | 96.19 | 1427 | [NR_025231.1](https://www.ncbi.nlm.nih.gov/nucleotide/NR_025231.1?report=genbank&log$=nucltop&blast_rank=8&RID=ASCN166V013) |
| *Amycolatopsis cihanbeyliensis* strain BNT52 16S ribosomal RNA, partial sequence | *Amycolatopsis cihanbeyliensis* | 1155 | 1155 | 100% | 0 | 96.19 | 1460 | [NR_109530.1](https://www.ncbi.nlm.nih.gov/nucleotide/NR_109530.1?report=genbank&log$=nucltop&blast_rank=9&RID=ASCN166V013) |
| *Amycolatopsis sacchari* strain NBRC 100339 16S ribosomal RNA, partial sequence | *Amycolatopsis sacchari* | 1149 | 1149 | 100% | 0 | 96.04 | 1445 | [NR_112696.1](https://www.ncbi.nlm.nih.gov/nucleotide/NR_112696.1?report=genbank&log$=nucltop&blast_rank=10&RID=ASCN166V013) |


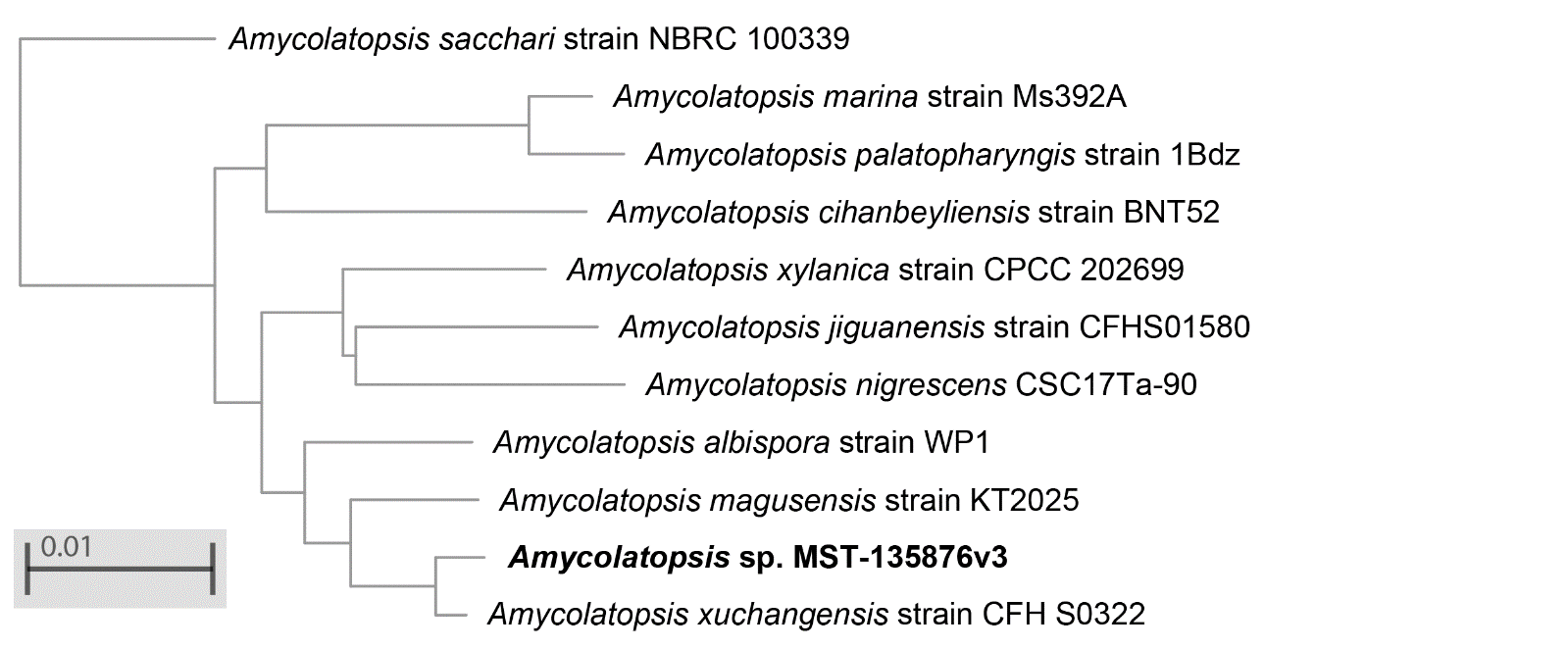


**Figure S2.** Phylogenetic relationships of *Amycolatopsis* sp. MST-135876v3. The phylogenetic tree was generated using NCBI Tree View.

135876v3 16S rRNA gene sequence. GenBank accession number: OK487575.

CATGCAAGTCGAACGATGAAGCCCTTCGGGGTGGATTAGTGGCGAACGGGTGAGTAACAC

GTGGGTAATCTGCCCTGTACTTTGGGATAAGCCCTGGAAACGGGGTCTAATACCGGATAT

GACTGCGCATCGCATGGTGTGTGGTGGAAAGCTCCGGCGGTATGGGATGAACCCGCGGCC

TATCAGCTTGTTGGTGGGGTAATGGCCCACCAAGGCGACGACGGGTAGCCGGCCTGAGAG

GGCGACCGGCCACACTGGGACTGAGACACGGCCCAGACTCCTACGGGAGGCAGCAGTGGG

GAATATTGCACAATGGGCGCAAGCCTGATGCAGCGACGCCGCGTGAGGGATGACGGCCTT

CGGGTTGTAAACCTCTTTCGACAGGGACGAAGGGTGACTGACGGTACCTGTAGAAGAAGC

ACCGGCTAACTACGTGCCAGCAGCCGCGGTAATACGTAGGGTGCGAGCGTTGTCCGGAAT

TATTGGGCGTAAAGAGCTCGTAGGCGGTTTGTCACGTCGGCTGTGAAAACTGGAGGCTTA

ACCTTCAGCTTGCAGTCGATACGGGCAGACTTGAGTTCGGTAGGGGAGACTGGAATTCCT

GGTGTAGCGGTGAAATGCGCAGATATCAGGAGGAACACCGGTGGCGAAGGCGGGTCTCTG

GGCCGATACTGACGCTGAGGAGCGAAAGCGTGGGGAGCGAACAGG

|  |  |
| --- | --- |

**Table S2.** ^1^H (600 MHz), ^13^C (150 MHz), HMBC, COSY, and ROESY NMR data for suertide A (**1**) in DMSO-*d_6_*.

|  | Pos. | δ_C_ | δ_H_ (*J* in Hz) | HMBC | COSY | ROESY |
| --- | --- | --- | --- | --- | --- | --- |
| d-Ser | NH |  | 8.19, d (6.1) | α, β, CO-Glu | α | β, α-Glu, βb-Glu, γ-Glu |
|  | CO | 170.3 |  |  |  |  |
|  | α | 56.2 | 4.12, q (6.5) | CO, β | NH, β | NH, β, NH-5-Cl-Trp |
|  | β | 60.3 | 3.27, m | CO, α | α | NH, α |
|  | OH |  | - |  |  |  |
| 5-Cl-d-Trp | NH |  | 8.66, d (8.2) | α, β, CO-Ser | α | βb, NH-6-Cl-Trp, α-Ser |
|  | CO | 170.8 |  |  |  |  |
|  | α | 54.2 | 4.26, m | CO, β, 3 | NH, βa/b | β, 1, 7 |
|  | βa | 26.9 | 3.20, dd (15.2, 3.7) | α, 2, 3, 3a | α, βb | α, βb, 1 |
|  | βb |  | 2.90, dd (15.2, 10.8) | α, 2, 3, 3a | α, βa | α, βa, 4 |
|  | 1 |  | 11.00, d (2.2) |  | 2 | 2 |
|  | 2 | 125.3 | 7.20, d (2.2) | 3, 3a, 7a | 1 | α, β, 1 |
|  | 3 | 110.6 |  |  |  |  |
|  | 3a | 128.3 |  |  |  |  |
|  | 4 | 117.4 | 7.57^a^, m | 5, 6, 7a |  | NH, α, βa/b |
|  | 5 | 123.1 |  |  |  |  |
|  | 6 | 120.8 | 7.04, dd (8.6, 2.0) | 4, 7a | 7 | 7 |
|  | 7 | 112.8 | 7.33^b^, m | 3a, 5 | 6 | 1, 6 |
|  | 7a | 134.5 |  |  |  |  |
| 6-Cl-d-Trp | NH |  | 7.37, d (2.0) | α, CO-5-Cl-Trp | α | α, βa |
|  | CO | 170.0 |  |  |  |  |
|  | α | 53.5 | 4.72, m | CO, β, 3 | NH, βa/b | NH, βa/b, NH-Ile |
|  | β | 28.6 | 3.07, dd (14.0, 8.6) | CO, α, 2, 3, 3a | α | α, 2, 4 |
|  |  |  | 2.98, dd (14.0, 4.7) | CO, α, 2, 3, 3a | α | α, 2, 4 |
|  | 1 |  | 11.00, d (1.9) | 2, 3, 3a, 7a | 2 | 2, 7 |
|  | 2 | 124.6 | 7.09, d (2.3) | 3, 3a, 7a | 1 | α, β, 1 |
|  | 3 | 109.9 |  |  |  |  |
|  | 3a | 126.5 |  |  |  |  |
|  | 4 | 120.1 | 7.56^a^, m | 6, 7a | 5 | α, βa, 5 |
|  | 5 | 118.5 | 6.98, dd (8.5, 1.9) | 3a, 7 | 4 | 4 |
|  | 6 | 125.5 |  |  |  |  |
|  | 7 | 110.7 | 7.33^b^, m | 3a, 5, 6 |  | 1 |
|  | 7a | 136.4 |  |  |  |  |
| l-Ile | NH |  | 8.21, d (7.6) | α, β, CO-6-Cl-Trp | α | β, γ2, α-Val, α-6-Cl-Trp |
|  | CO | 172.2 |  |  |  |  |
|  | α | 57.9 | 4.00, dd (8.8, 7.6) | CO, β, γ1/2 | NH, β | β, γ1a/b, γ2 |
|  | β | 34.9 | 1.50, m |  | α, γ2 | NH, α, γ1a, γ2, δ |
|  | γ1a | 25.0 | 1.17, m | α, β, γ2, δ | δ | α, γ1b, γ2 |
|  | γ1b |  | 0.91, m | α, β, γ2, δ | δ | α, γ1a, γ2 |
|  | γ2 | 14.6 | 0.47, d (6.8) | α, β, γ1 | β | α, β, δ |
|  | δ | 10.7 | 0.73, t (7.4) | β, γ1 | γ1a/b | α, β, γ1b |
| d-Val | NH |  | 8.34, d (8.8) | α, β, CO-Ile | α | α, β, γ1/2, α-Glu |
|  | CO | 170.4 |  |  |  |  |
|  | α | 58.0 | 4.07, dd (8.8, 4.5) | CO, β, γ2, CO-Ile | β | NH, β, γ1/2 |
|  | β | 29.0 | 2.26, m | γ1/2 | γ1/2 | NH, α, γ1/2 |
|  | γ1 | 19.4 | 0.81, d (6.9) | α, β, γ2 | β | NH, α, β |
|  | γ2 | 17.0 | 0.82, d (6.9) | α, β, γ1 | β | NH, α, β |
| d-Glu | NH |  | 7.28, m (7.5) | α, CO-Val | α | α, βa/b, γ, NH-Val, α-Val, γ1/2-Val |
|  | CO | 170.3 |  |  |  |  |
|  | α | 51.0 | 4.40, q (6.7) | CO, β, γ | NH, α, γa/b | NH, βa/b, γ, NH-Ser |
|  | βa | 28.5 | 1.89, m | CO, α, γ, δ | β, γ | NH, α, βb, γ, NH-Ser |
|  | βb |  | 1.79, m | CO, α, γ, δ | β, γ | NH, α, βa, γ, NH-Ser |
|  | γ | 29.7 | 2.18, t (8.3) | α, β, δ | βa/b | NH, α, βa/b, NH-Ser |
|  | δ | 173.9 |  |  |  |  |
|  | OH |  | 12.10, br s |  |  |  |

^a, b^ Overlapping resonances

|  |  |
| --- | --- |

**Table S3.** ^1^H (600 MHz), ^13^C (150 MHz), HMBC, COSY, and ROESY NMR data for suertide B (**2**) in DMSO-*d_6_*.

| Unit | Pos. | δ_C_ | δ_H_ (*J* in Hz) | HMBC | COSY | ROESY |
| --- | --- | --- | --- | --- | --- | --- |
| d-Ser | NH |  | 8.19, d (5.7) | α, β, CO-Glu | α | β, α-Glu |
|  | CO | 170.3 |  |  |  |  |
|  | α | 56.3 | 4.12, m | CO, β | NH, β | NH, β, NH-Trp |
|  | β | 60.4 | 3.28, m | CO, α | α | NH, α |
|  | OH |  |  |  |  |  |
| d-Trp | NH |  | 8.65, d (8.0) | α, βa/b, CO-Ser | α | βb, NH-6-Br-Trp, α-Ser |
|  | CO | 170.9 |  |  |  |  |
|  | α | 54.3 | 4.26, m | CO, βa/b | NH, βa/b | βa, 2 |
|  | βa | 27.0 | 3.25, m | α, 2, 3, 3a | α, βb | α, βb |
|  | βb |  | 2.89, dd (15.0, 11.0) | α, 2, 3, 3a | α, βa | NH, βa |
|  | 1 | 10.8 |  |  | 2 | 2, 7 |
|  | 2 | 123.2 | 7.11^c^, m | 3, 3a, 7a | 1 | NH, α, βa/b, 1 |
|  | 3 | 110.6 |  |  |  |  |
|  | 3a | 127.1 |  |  |  |  |
|  | 4 | 118.0 | 7.53^b^, d (7.8) | 3, 7, 7a | 5 | 5 |
|  | 5 | 118.3 | 6.98, m | 3a, 6, 7 | 4, 6 | 4 |
|  | 6 | 120.9 | 7.04, m | 4, 7a | 5, 7 | 7 |
|  | 7 | 111.3 | 7.31, d (8.1) | 3a, 5 | 6 | 6 |
|  | 7a | 136.0 |  |  |  |  |
| 6-Br-d-Trp | NH |  | 7.37, d (7.1) | α, CO-Trp | α | α, NH-Trp |
|  | CO | 170.0 |  |  |  |  |
|  | α | 53.4 | 4.73, m | CO, β, 3 | NH, βa/b | βa/b, 2, 7, NH-Ile |
|  | βa | 28.6 | 3.09, dd (14.1, 8.4) | CO, α, 2, 3, 3a | α, βb | α |
|  | βb |  | 2.99, dd (14.1, 4.9) | CO, α, 2, 3, 3a | α, βa | α |
|  | 1 |  | 10.97, d (2.1) | 2, 3, 3a, 7a | 2 | 2, 7 |
|  | 2 | 124.5 | 7.09, m | 3, 3a, 7a | 1 | α, βa/b, 1 |
|  | 3 | 109.9 |  |  |  |  |
|  | 3a | 126.8 |  |  |  |  |
|  | 4 | 120.5 | 7.52^b^, d (5.2) | 6, 7a | 5 | 5 |
|  | 5 | 121.0 | 7.11^c^, m | 7 | 4 | 4 |
|  | 6 | 113.6 |  |  |  |  |
|  | 7 | 113.6 | 7.48, d (1.7) | 3a, 5, 6 |  | 1 |
|  | 7a | 136.8 |  |  |  |  |
| l-Ile | NH |  | 8.21, d (7.3) | α, β, CO-6Br | α | α, β, γ2, α-6-Br-Trp |
|  | CO | 172.2 |  |  |  |  |
|  | α | 57.9 | 4.00, dd (8.7, 7.6) | CO, γ1, γ2 | NH, β | β, γ2, δ, NH-Val |
|  | β | 34.9 | 1.51, m |  | α, γ1, γ2 | NH, α, γ1b, γ2, δ |
|  | γ1a | 24.9 | 1.19, m | α, β, γ2, δ | γ1b, δ | γ1b, δ |
|  | γ1b |  | 0.93, m | α, β, γ2, δ | γ1a, δ | γ1a, γ2 |
|  | γ2 | 14.6 | 0.49, d (6.9) | α, β, γ1 | β | NH, α, β, γ1b, δ |
|  | δ | 10.7 | 0.75, t (7.4) | α, β, γ1 | γ1a/b | α, β |
| d-Val | NH |  | 8.34, d (8.8) | α, CO-Ile | α | α, γ1, γ2 |
|  | CO | 170.4 |  |  |  |  |
|  | α | 58.0 | 4.07, dd (8.8, 4.5) | CO, β, γ2, CO-Ile | NH, β | NH, β, γ1, γ2 |
|  | β | 29.0 | 2.26, m | α, γ1, γ2 | γ1, γ2 | α, γ1, γ2 |
|  | γ1 | 19.4 | 0.82^a^, d (2.9) | α, β, γ2 | β | NH, α, β |
|  | γ2 | 16.9 | 0.82^a^, d (2.9) | α, β, γ1 | β | NH, α, β |
| d-Glu | NH |  | 7.27, d (7.8) | Α, CO-Val | α | α, γ, γ1/2-Val |
|  | CO | 170.3 |  |  |  |  |
|  | α | 51.0 | 4.40, q (6.7) | CO, β, γ | NH, β | βa/b, γ |
|  | βa | 28.4 | 1.89, m | CO, α, γ, δ | α, βb, γ | α, βb, γ |
|  | βb |  | 1.78, m | CO, α, γ, δ | α, βa, γ | α, βa, γ |
|  | γ | 29.7 | 2.18, t (8.3) | CO, α, βa/b, δ | βa/b | α, βa/b |
|  | δ | 173.9 |  |  |  |  |
|  | OH |  | 12.10, br s |  |  |  |

^a – c^ Overlapping signals

|  |  |
| --- | --- |

**Table S4.** ^1^H (600 MHz), ^13^C (150 MHz), HMBC, COSY, and ROESY NMR data for suertide C (**3**) in DMSO-*d_6_*.

|  | Pos. | δ_C_ | δ_H_ (*J* in Hz) | HMBC | COSY | ROESY |
| --- | --- | --- | --- | --- | --- | --- |
| l-Ser | NH |  | 8.20, m | α, β, CO-Glu | α | α, β, α-Glu |
|  | CO | 170.4 |  |  |  |  |
|  | α | 56.2 | 4.11, q | CO, β | NH, β | NH, β, NH-5-Br-Trp |
|  | β | 60.3 | 3.26, m | CO, α | α | α, NH |
|  | OH |  |  |  |  |  |
| 5-Br-d-Trp | NH |  | 8.66, d (8.1) | α, βa/b, CO-Ser | α | α, βb, 2, α-Ser |
|  | CO | 170.8 |  |  |  |  |
|  | α | 54.2 | 4.25, m | CO, β | βa/b | NH, βa/b, 2, 4 |
|  | βa | 26.8 | 3.21, dd (15.0, 2.9) | α, 2, 3, 3a | α, βa |  |
|  | βb |  | 2.90, dd (15.0, 10.8) | CO, α, 2, 3, 3a | α, βb |  |
|  | 1 |  | 11.00, d (1.9) | 2, 3, 3a, 7a | 2 | 2, 7 |
|  | 2 | 125.1 | 7.19, d (2.3) | 3, 3a, 7a | 1 | NH, α, βa/b, 1 |
|  | 3 | 110.5 |  |  |  |  |
|  | 3a | 129.0 |  |  |  |  |
|  | 4 | 120.4 | 7.71, d (1.9) | 5, 6, 7a | 6 | NH, α, βa/b |
|  | 5 | 111.0 |  |  |  |  |
|  | 6 | 123.3 | 7.15, dd (8.6, 1.9) | 4, 7, 7a | 7 | 7 |
|  | 7 | 113.3 | 7.29, d (8.6) | 3a, 5, 6 | 6 | NH-Val, α-Glu, α-Val, γ-Glu, γ1-Val |
|  | 7a | 134.7 |  |  |  |  |
| 6-Br-d-Trp | NH |  | 7.37, d (7.1) | α, CO-5Br | α | α |
|  | CO | 170.0 |  |  |  |  |
|  | α | 53.5 | 4.73, m | CO, β, 3 | NH, βa/b | βa/b, 2, 4, NH-Ile |
|  | βa | 28.4 | 3.07, dd (14.0, 8.6) | CO, α, 2, 3, 3a | α | α, βb, 4 |
|  | βb |  | 2.98, dd (14.0, 4.8) | CO, α, 2, 3, 3a | α | α, βa, 4 |
|  | 1 |  | 11.00, d (1.9) | 2, 3a, 7a | 2 | 2, 3a |
|  | 2 | 124.6 | 7.08, d (2.3) | 3, 3a, 7a | 1, 4 | βa, 1, NH-Ile |
|  | 3 | 109.9 |  |  |  |  |
|  | 3a | 126.7 |  |  |  |  |
|  | 4 | 120.5 | 7.51, d (8.4) | 3, 6, 7a | 5 | α, βa/b, 5 |
|  | 5 | 121.0 | 7.10, dd (8.4, 1.7) | 3a, 7 | 4 | 4 |
|  | 6 | 113.6 |  |  |  |  |
|  | 7 | 113.5 | 7.48, d (1.7) | 3a, 5, 6 |  | 1 |
|  | 7a | 136.8 |  |  |  |  |
| l-Ile | NH |  | 8.20, m | α, β, CO-6Cl | α | α, β, γ2 |
|  | CO | 172.2 |  |  |  |  |
|  | α | 57.9 | 4.00, dd (8.7, 7.6) | CO, β, γ1, γ2, CO-6Br | NH, β | NH, β, γ1a/b, γ2, δ, NH-Val |
|  | β | 34.9 | 1.50, m |  | α, γ1b, γ2 | NH, α, γ1a, δ |
|  | γ1a | 24.9 | 1.18, m | α, β, γ, δ | γ1b, δ | β, γ1b, γ2, δ |
|  | γ1b |  | 0.92, m | α, β, γ, δ | β, γ1a, δ | α, β, γ1a, γ2 |
|  | γ2 | 14.6 | 0.47, d (6.8) | α, β, γ1 | β | NH, α, β, γ1a/b, δ, C2-6-Br-Trp |
|  | δ | 10.7 | 0.73, t (7.4) | β, γ1 | γ1a/b | α, β, γ1a/b, γ2 |
| d-Val | NH |  | 8.35, d (8.8) | α, β, CO-Ile | α | α, β, γ1, γ2, NH-Glu, α-Ile |
|  | CO | 170.3 |  |  |  |  |
|  | α | 58.0 | 4.07, dd (8.8, 4.5) | CO, β, γ2, CO-Ile | NH, β | NH, β, γ1, γ2 |
|  | β | 28.6 | 2.26, m | α, γ1, γ2 | α, γ1, γ2 | NH, α, γ1, γ2 |
|  | γ1 | 19.4 | 0.81^a^, d (2.9) | α, β | β | NH, α, β |
|  | γ2 | 16.9 | 0.83^a^, d (2.9) | α, β | β | NH, α, β |
| l-Glu | NH |  | 7.27, d (7.6) | α, CO-Val | α | α, βa/b, γ, α-Ser |
|  | CO | 170.3 |  |  |  |  |
|  | α | 51.0 | 4.41, d (6.7) | CO, βa/b, γ | NH, βa/b | βa/b, γ, NH-Ser |
|  | βa | 29.0 | 1.88, m | CO, α, γ, δ | α, βa | α, γ, NH-Ser |
|  | βb |  | 1.80, m | CO, α, γ, δ | α, βb | α, γ, NH-Ser |
|  | γ | 29.7 | 2.18, t (8.3) | CO, δ | βa/b | NH, α, βa/b, NH-Ser |
|  | δ | 173.9 |  |  |  |  |
|  | OH |  | 12.09, br s |  |  |  |

^a^ Overlapping signals


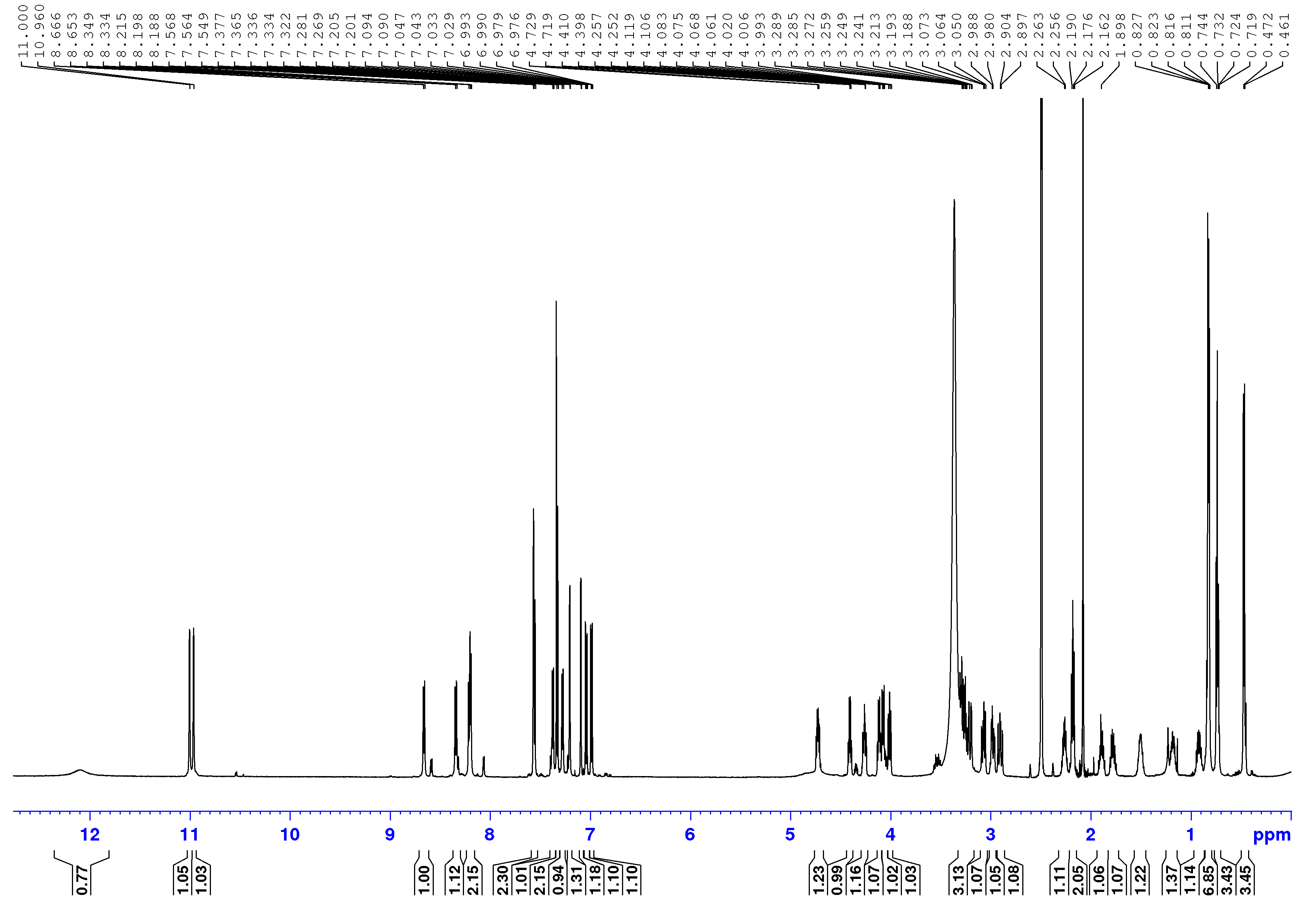


**Figure S3.** ^1^H NMR spectrum (600 MHz, DMSO-*d*_6_) of suertide A (**1**).


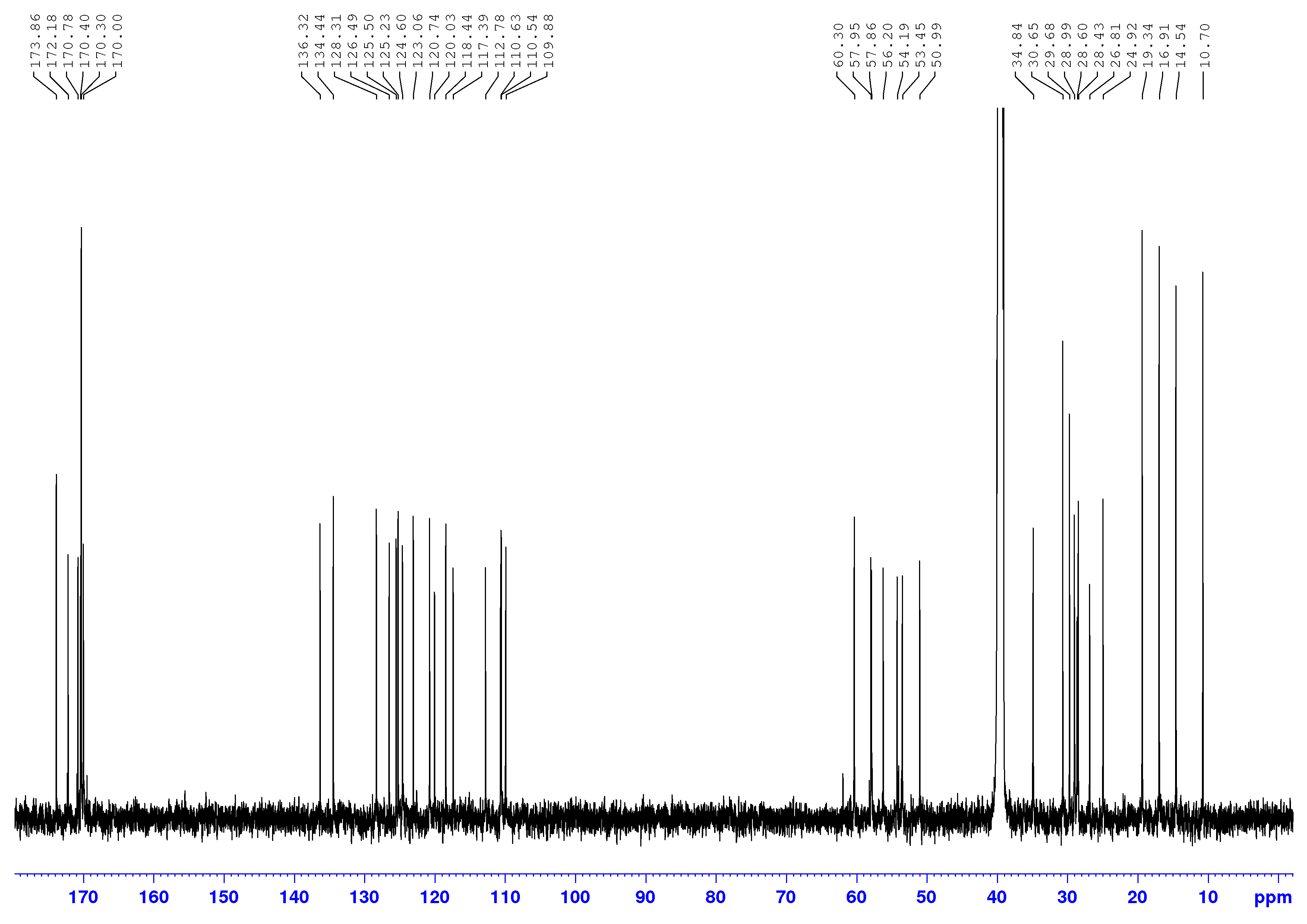


**Figure S4.** ^13^C NMR spectrum (150 MHz, DMSO-*d*_6_) of suertide A (**1**).


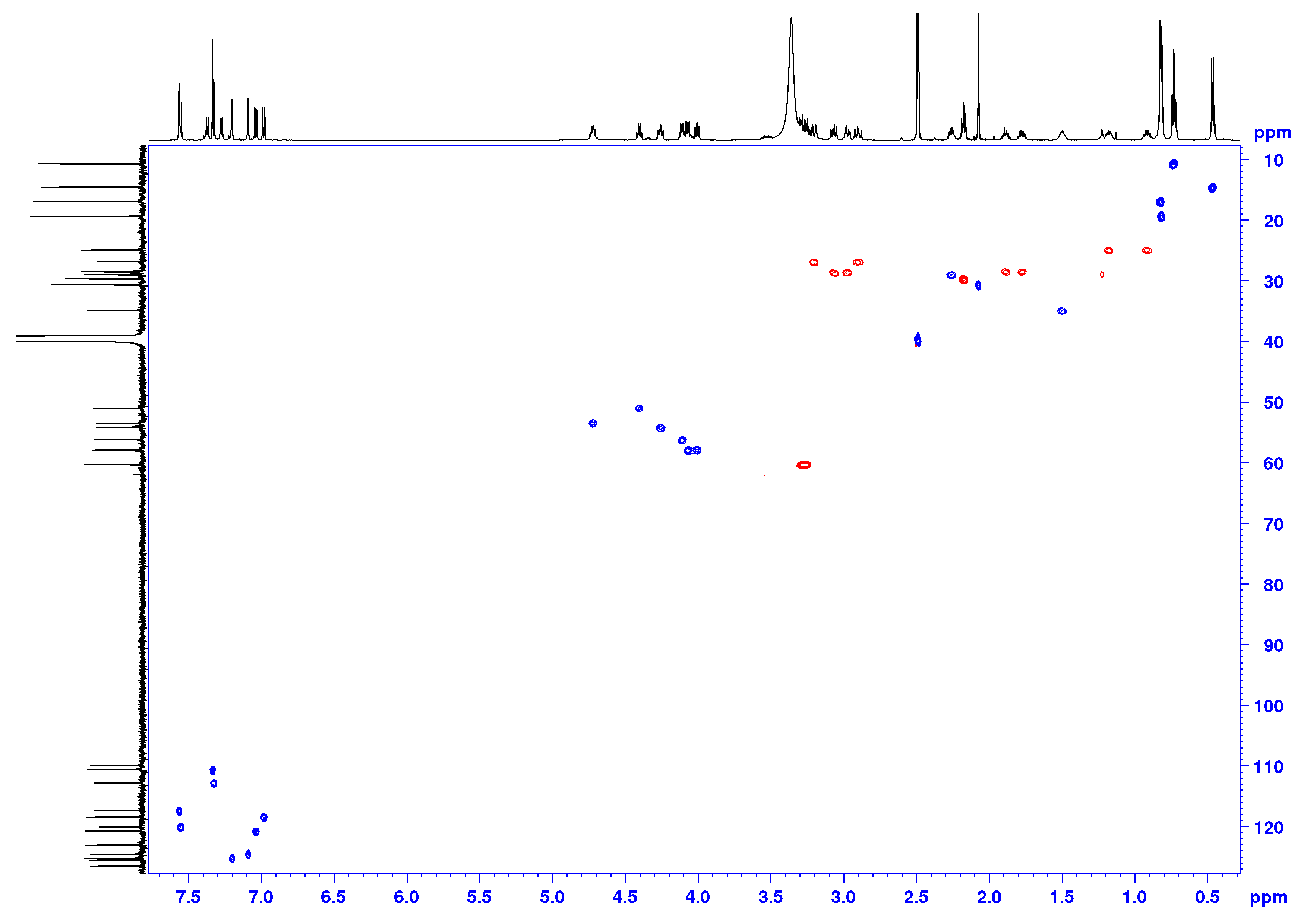


**Figure S5**. HSQC NMR spectrum (600 MHz, DMSO-*d*_6_) of suertide A (**1**).


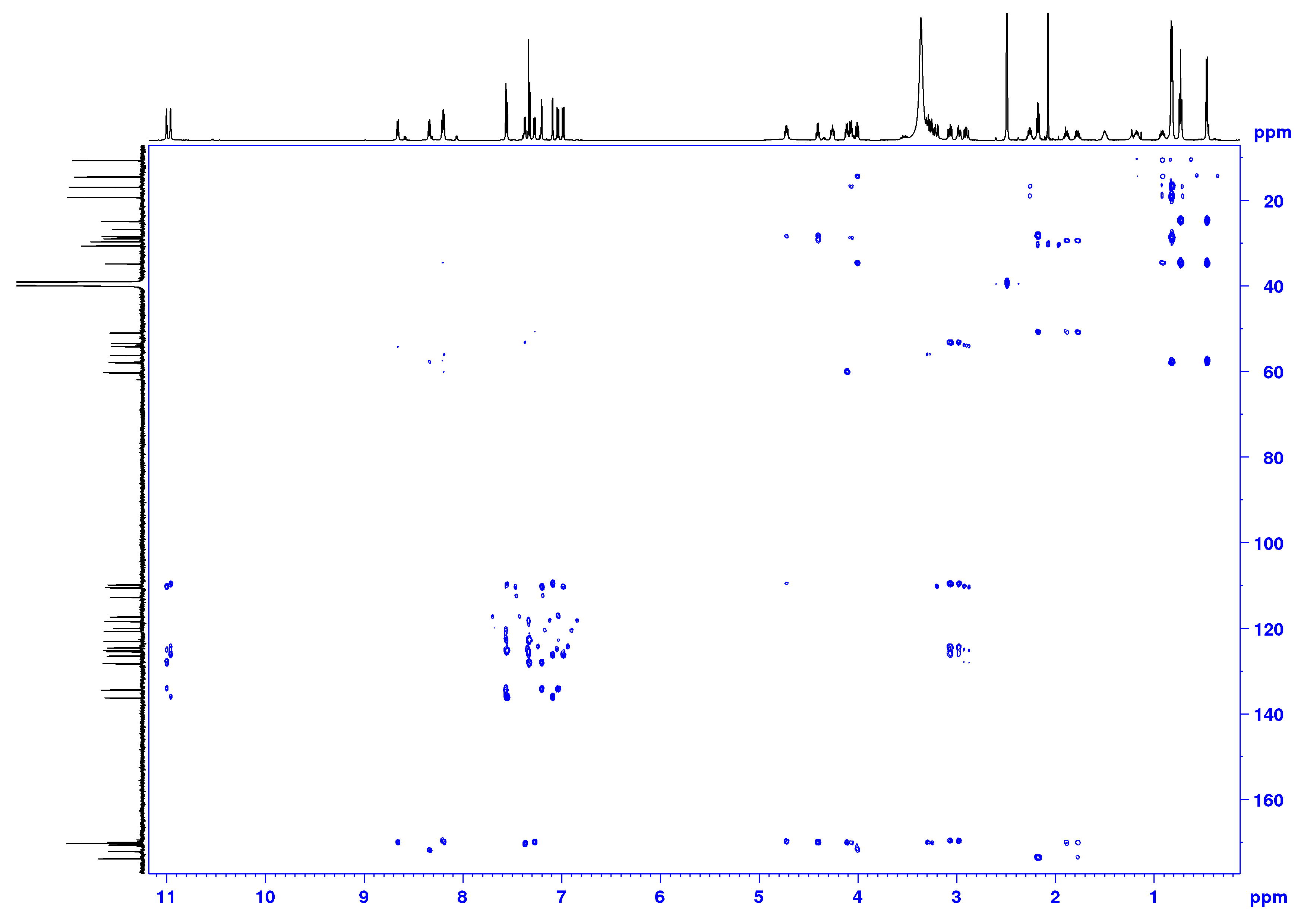


**Figure S6.** HMBC NMR spectrum (600 MHz, DMSO-*d*_6_) of suertide A (**1**).


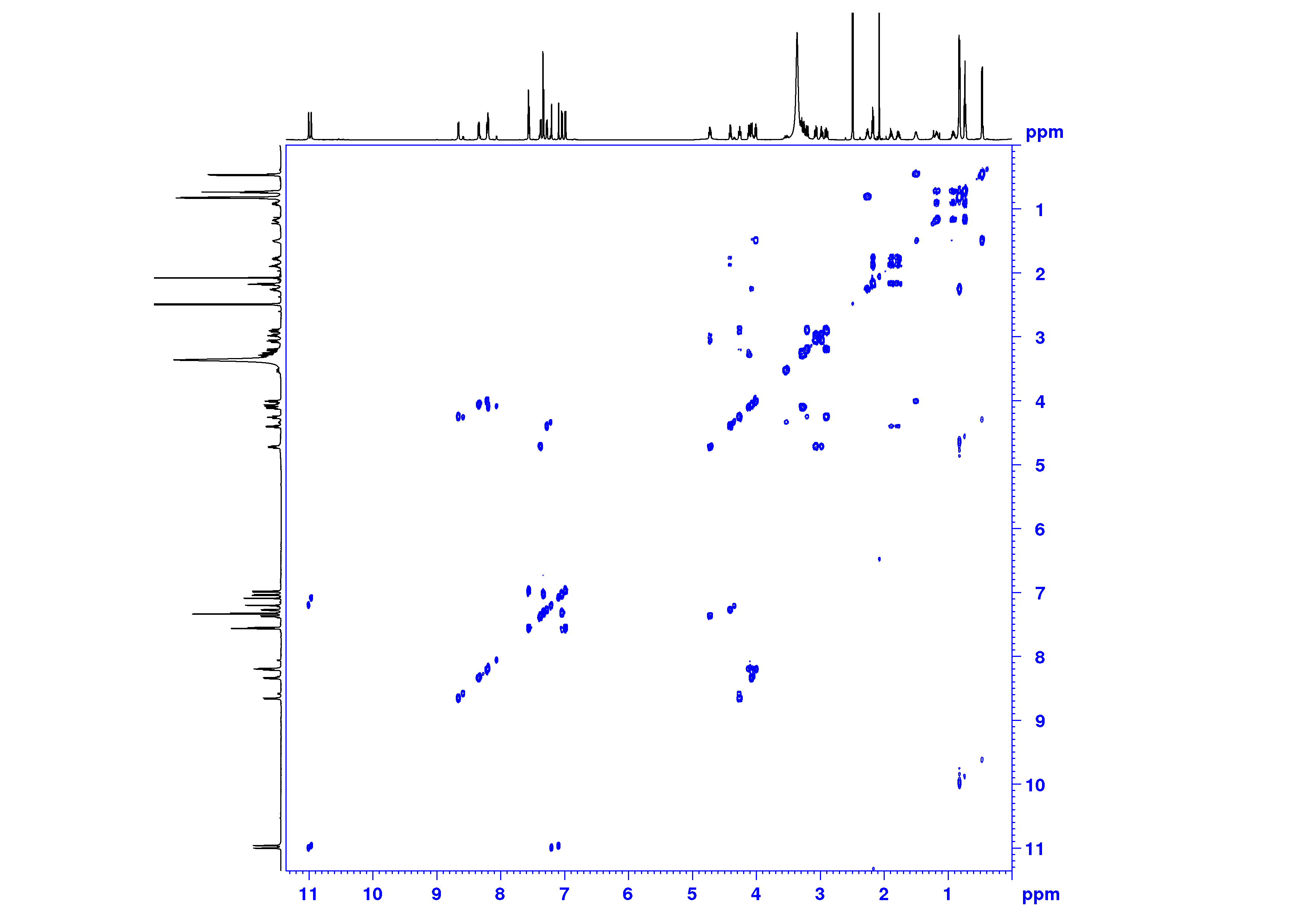


**Figure S7.** COSY NMR spectrum (600 MHz, DMSO-*d*_6_) of suertide A (**1**).


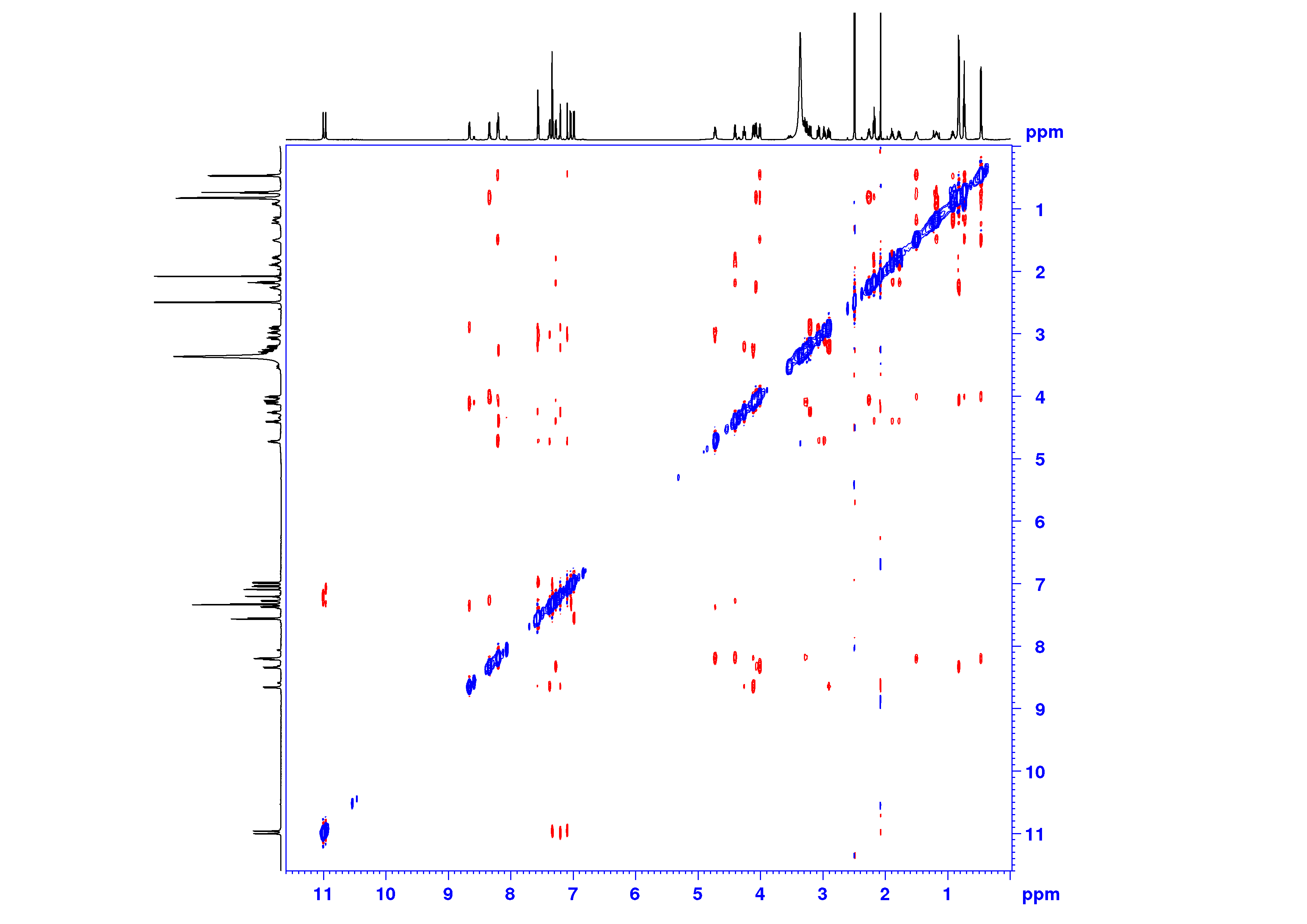


**Figure S8.** ROESY NMR spectrum (600 MHz, DMSO-*d*_6_) of suertide A (**1**).


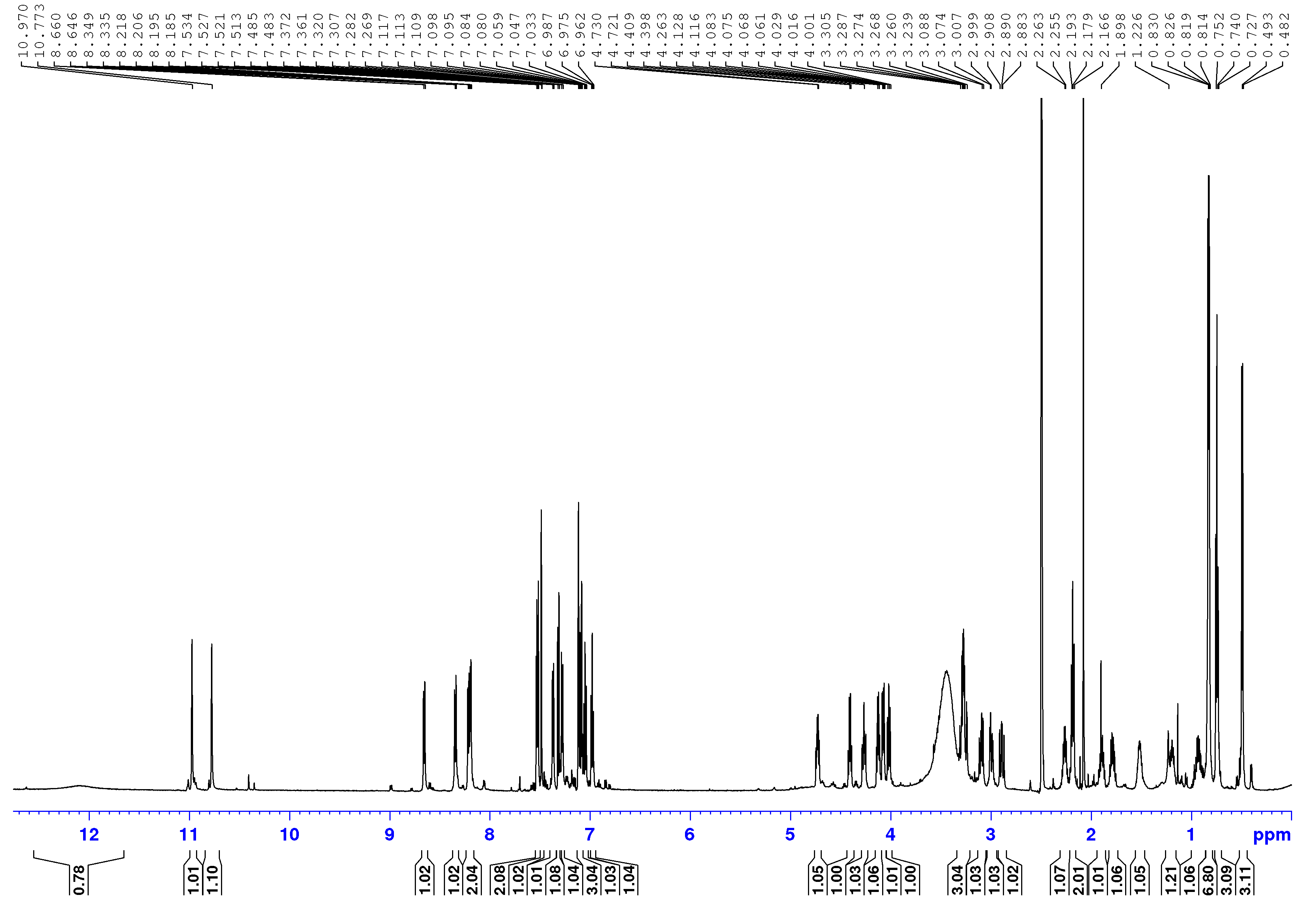


**Figure S9**. ^1^H NMR spectrum (600 MHz, DMSO-*d*_6_) of suertide B (**2**).


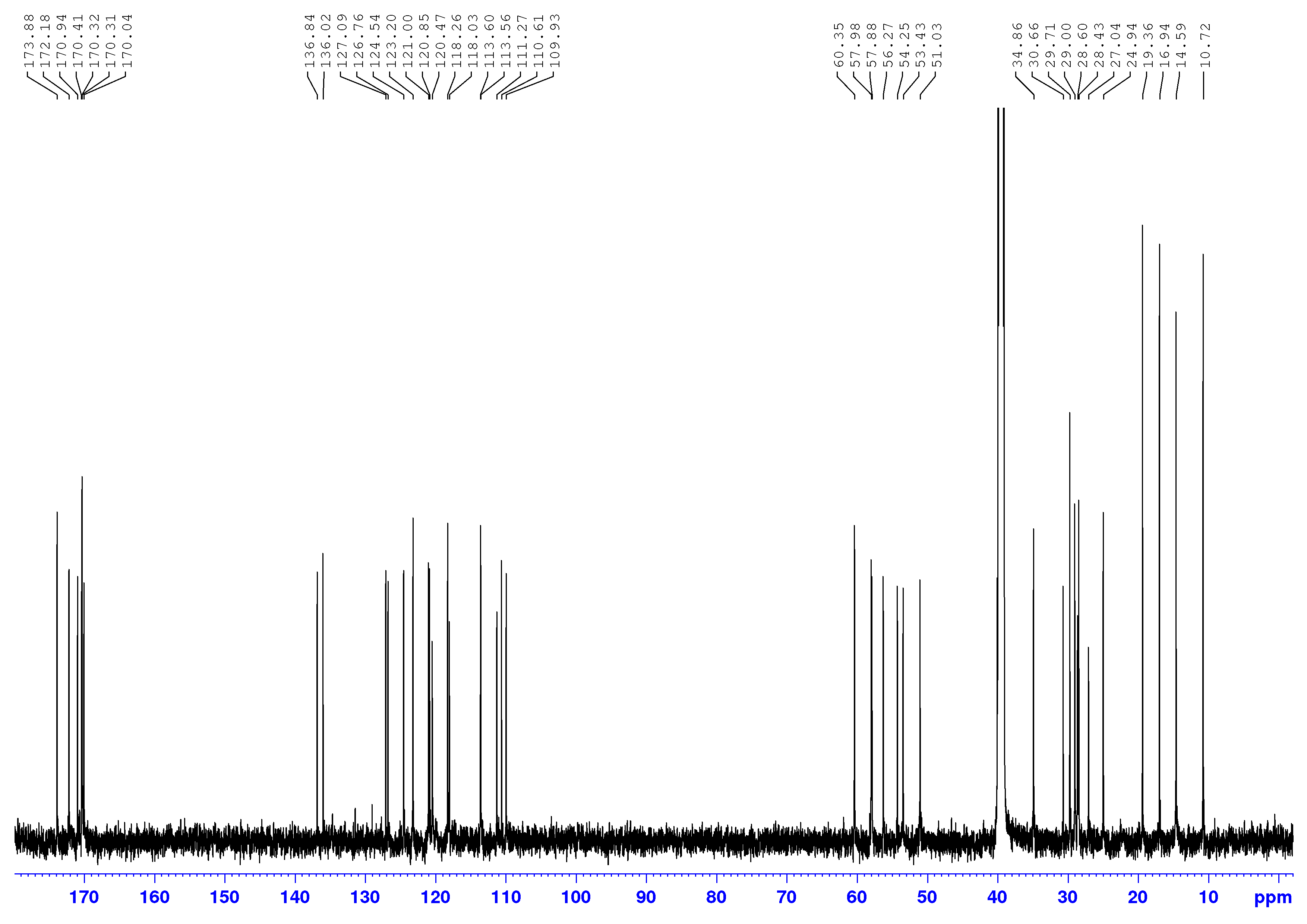


**Figure S10.** ^13^C NMR spectrum (150 MHz, DMSO-*d*_6_) of suertide B (**2**).


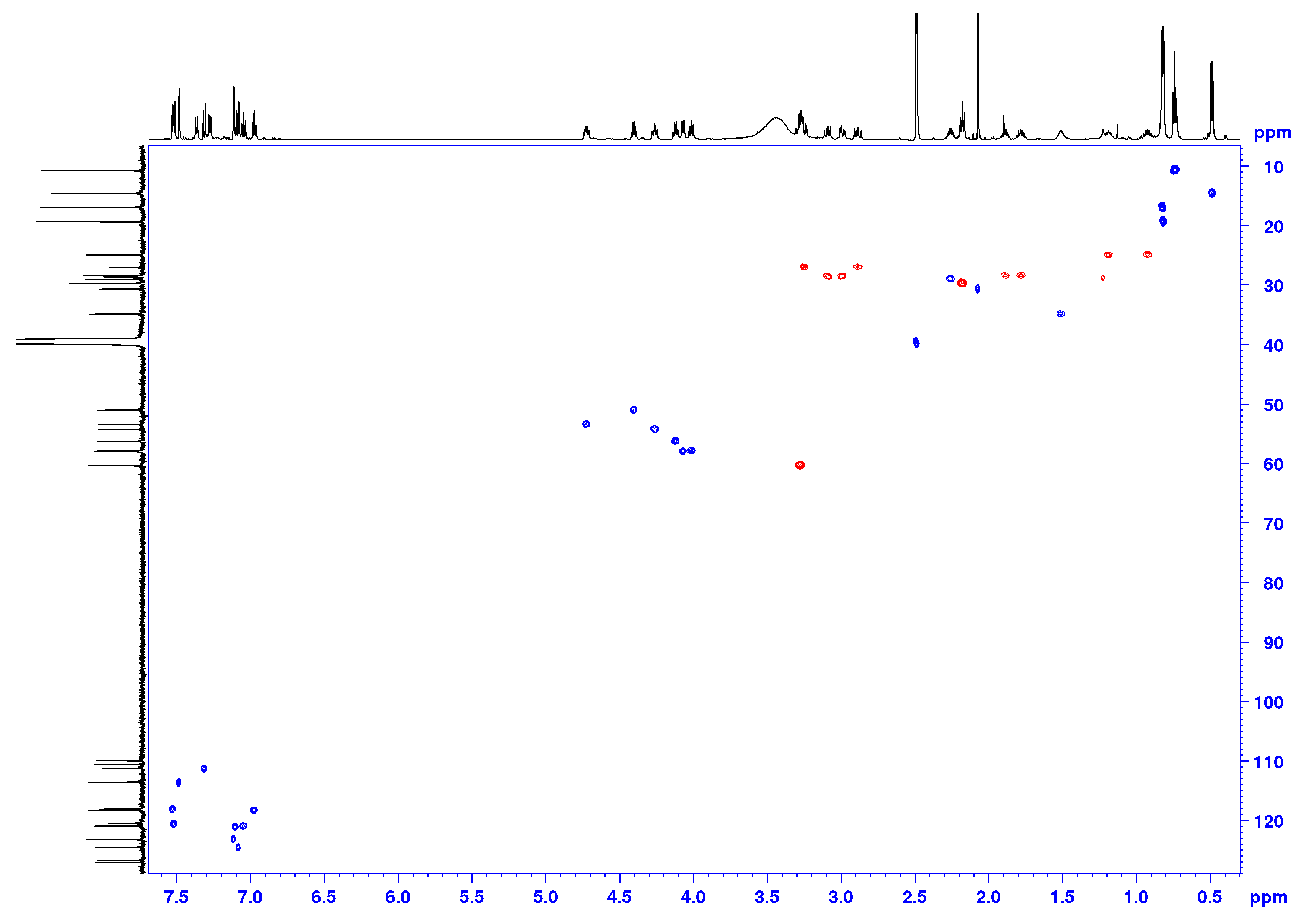


**Figure S11.** HSQC NMR spectrum (600 MHz, DMSO-*d*_6_) of suertide B (**2**).


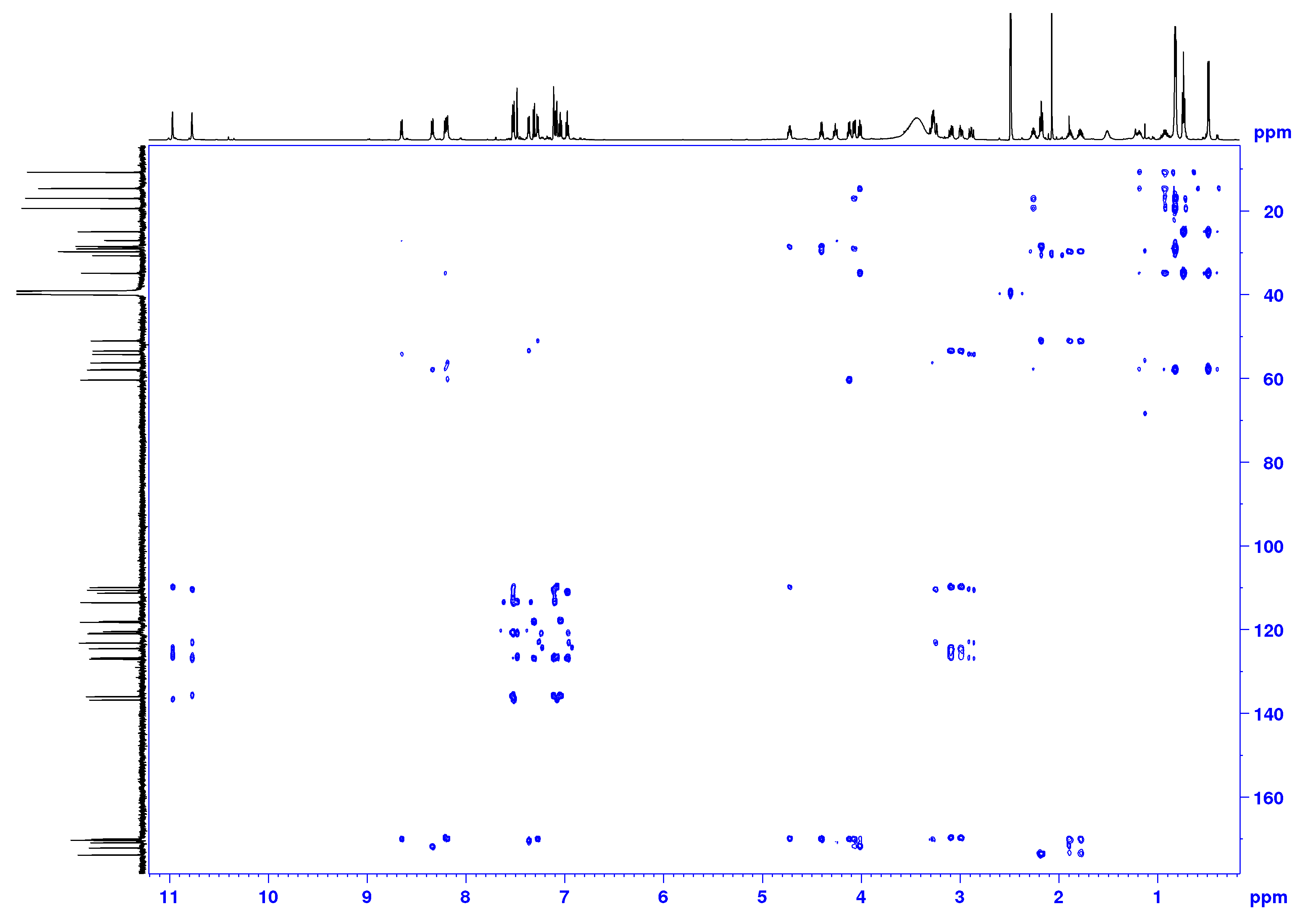


**Figure S12.** HMBC NMR spectrum (600 MHz, DMSO-*d*_6_) of suertide B (**2**).


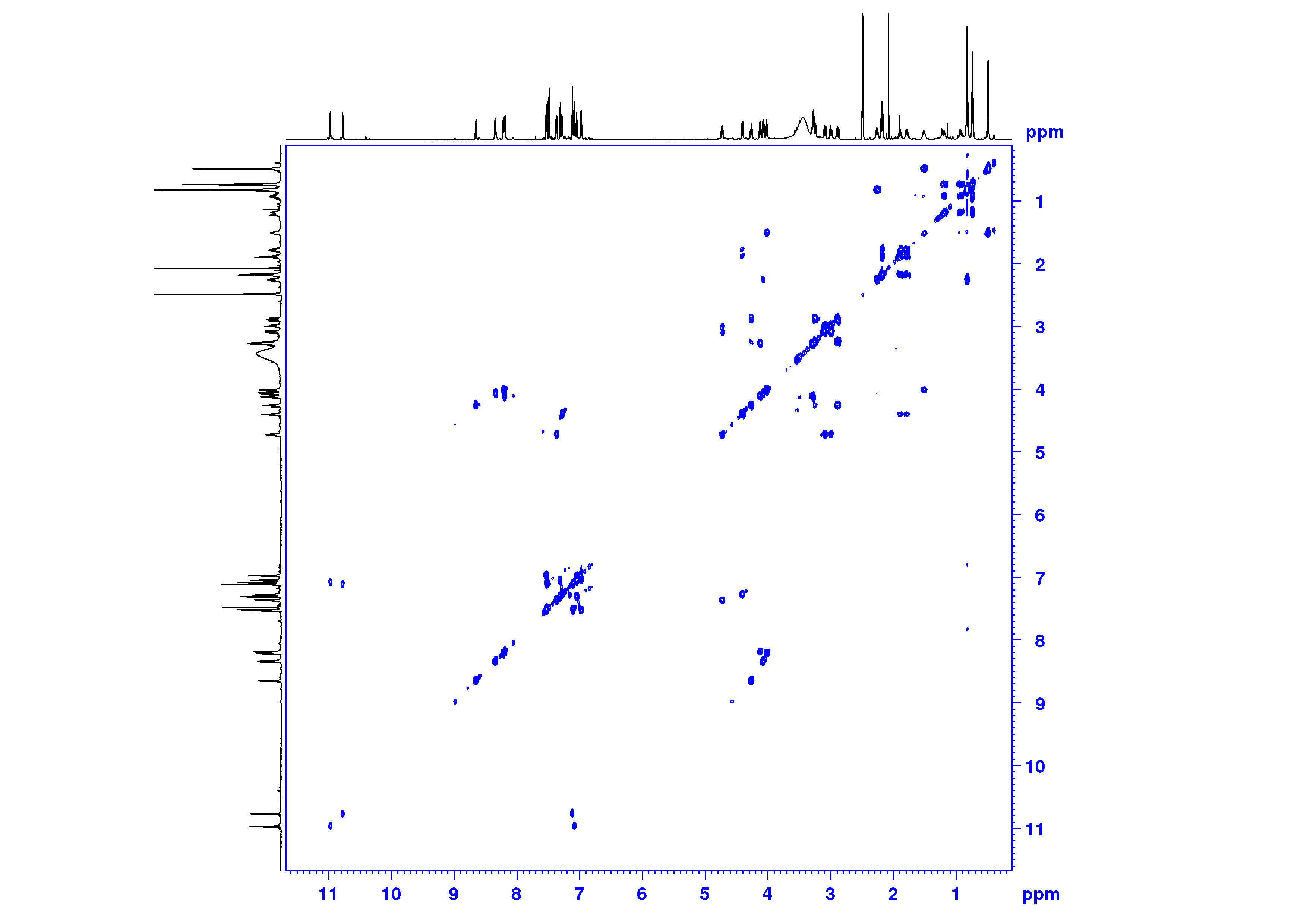


**Figure S13.** COSY NMR spectrum (600 MHz, DMSO-*d*_6_) of suertide B (**2**).


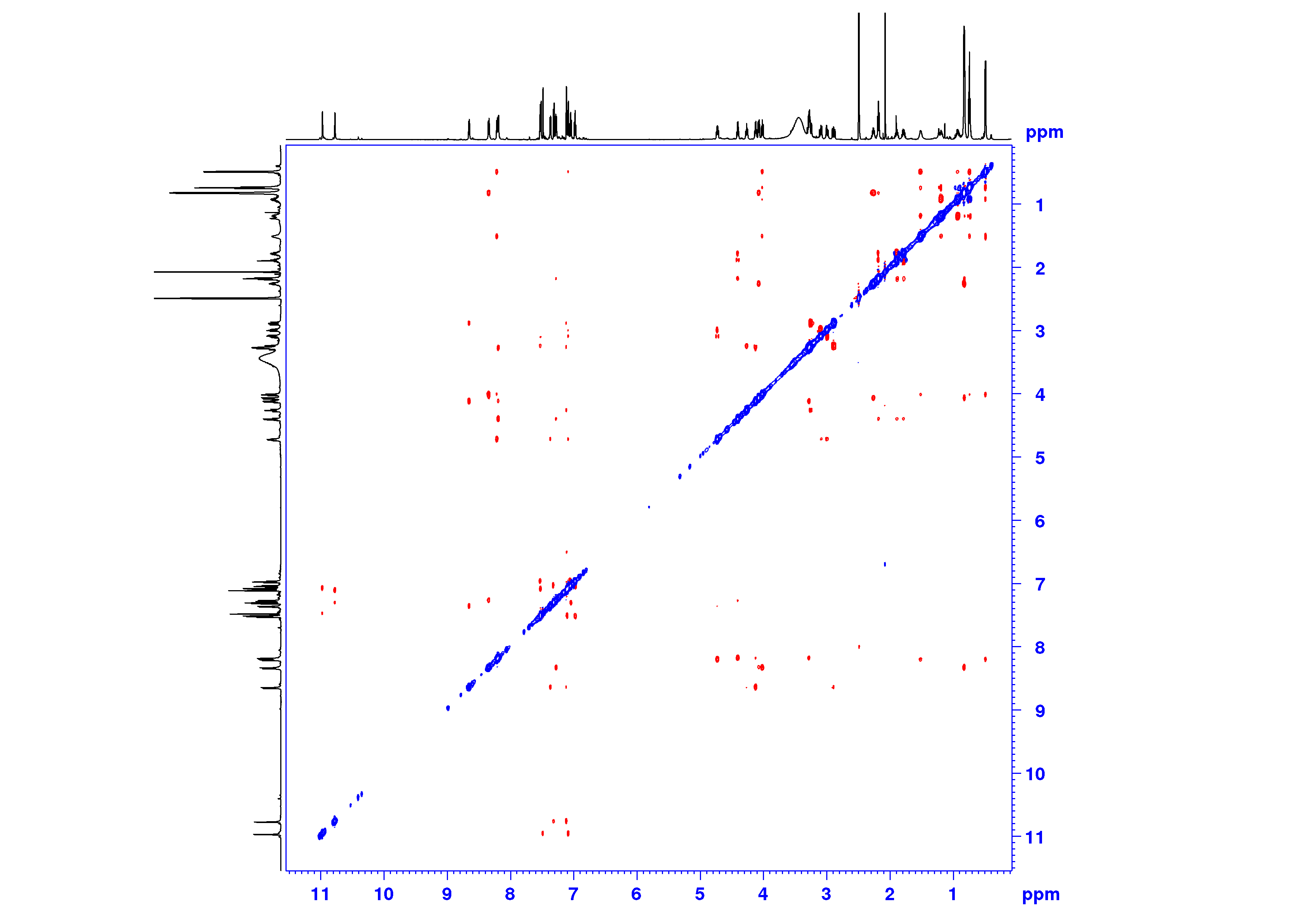


**Figure S14.** ROESY NMR spectrum (600 MHz, DMSO-*d*_6_) of suertide B (**2**).


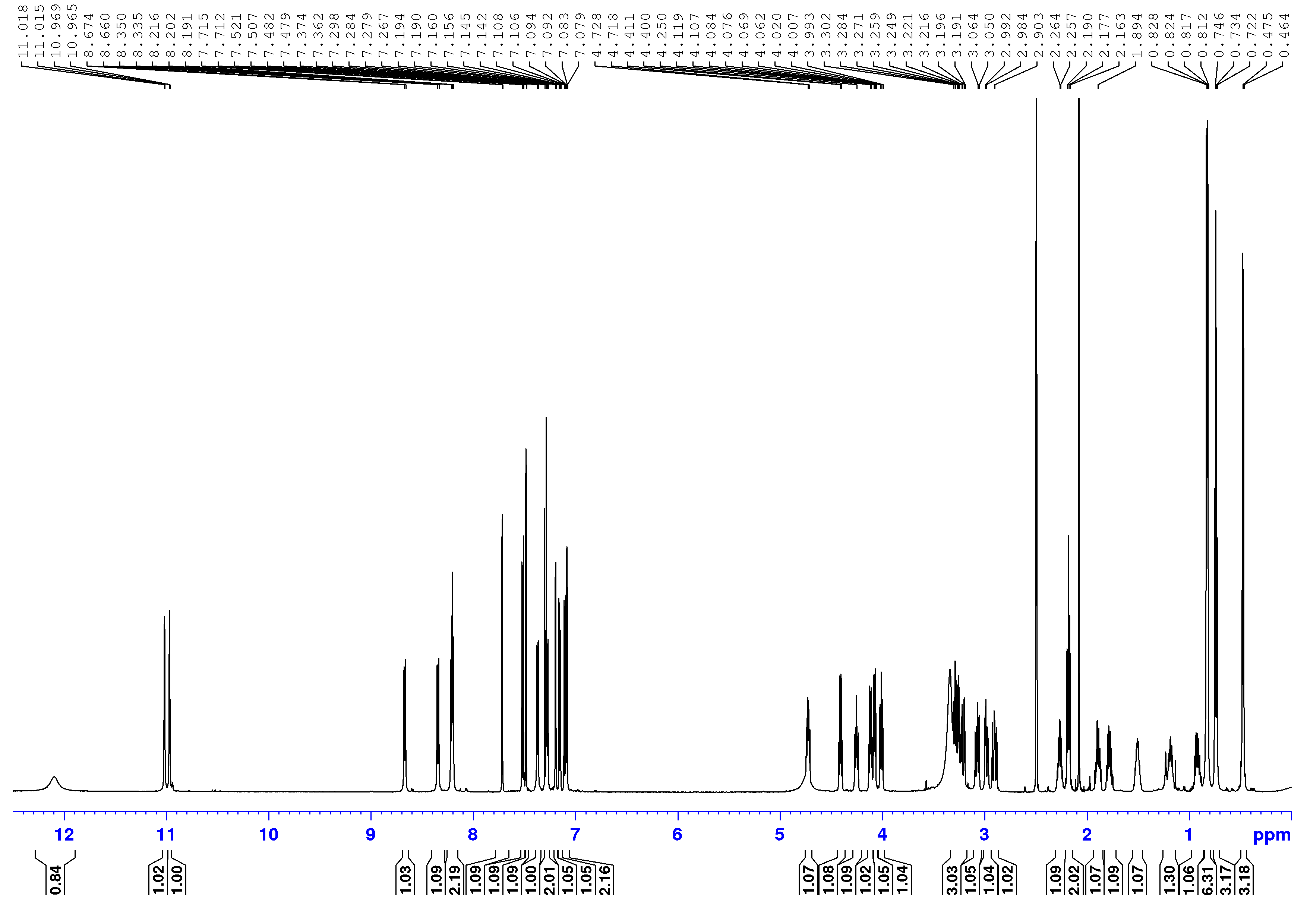


**Figure S15**. ^1^H NMR spectrum (600 MHz, DMSO-*d*_6_) of suertide C (**3**).


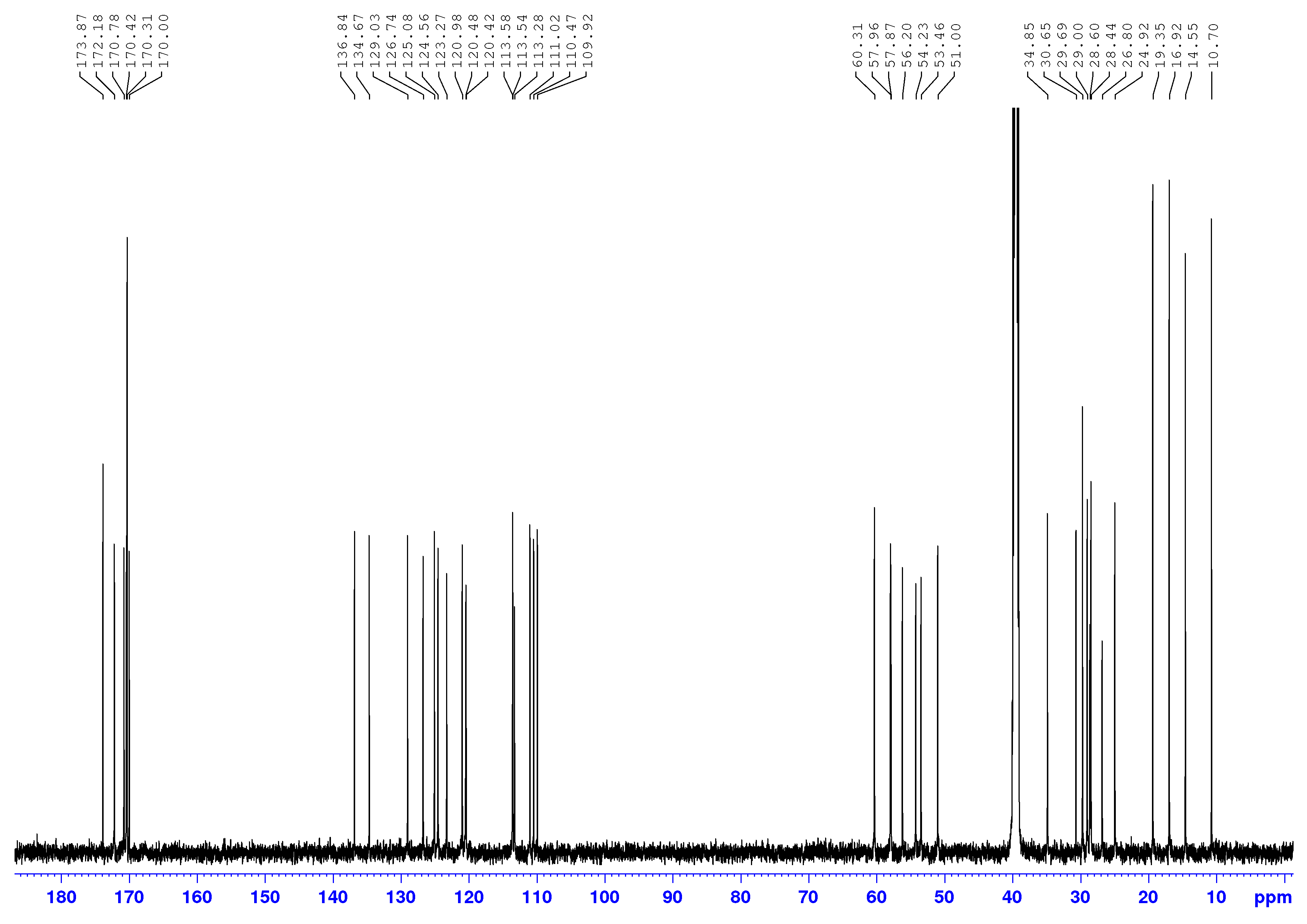


**Figure S16.** ^13^C NMR spectrum (150 MHz, DMSO-*d*_6_) of suertide C (**3**).


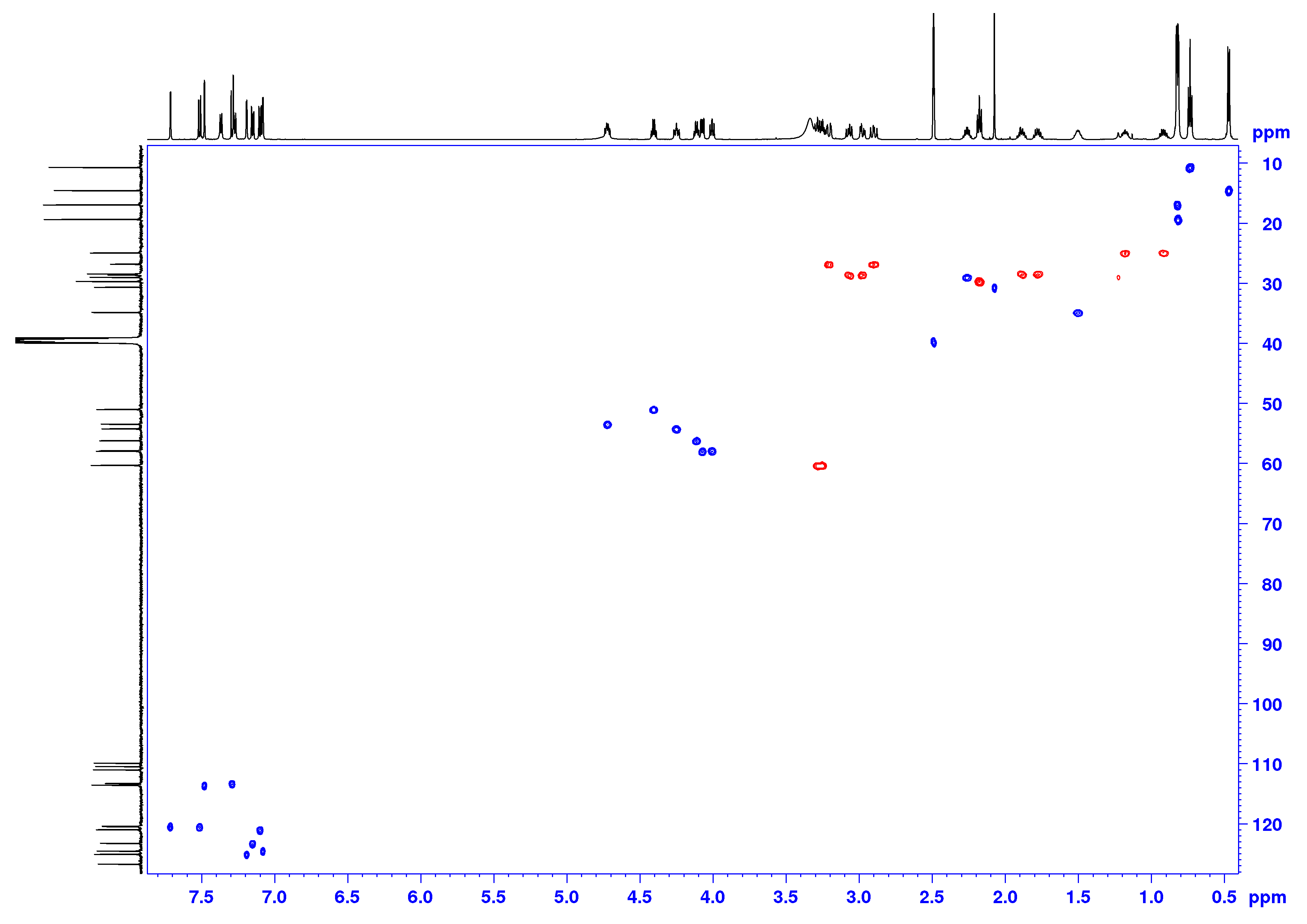


**Figure S17.** HSQC NMR spectrum (600 MHz, DMSO-*d*_6_) of suertide C (**3**).


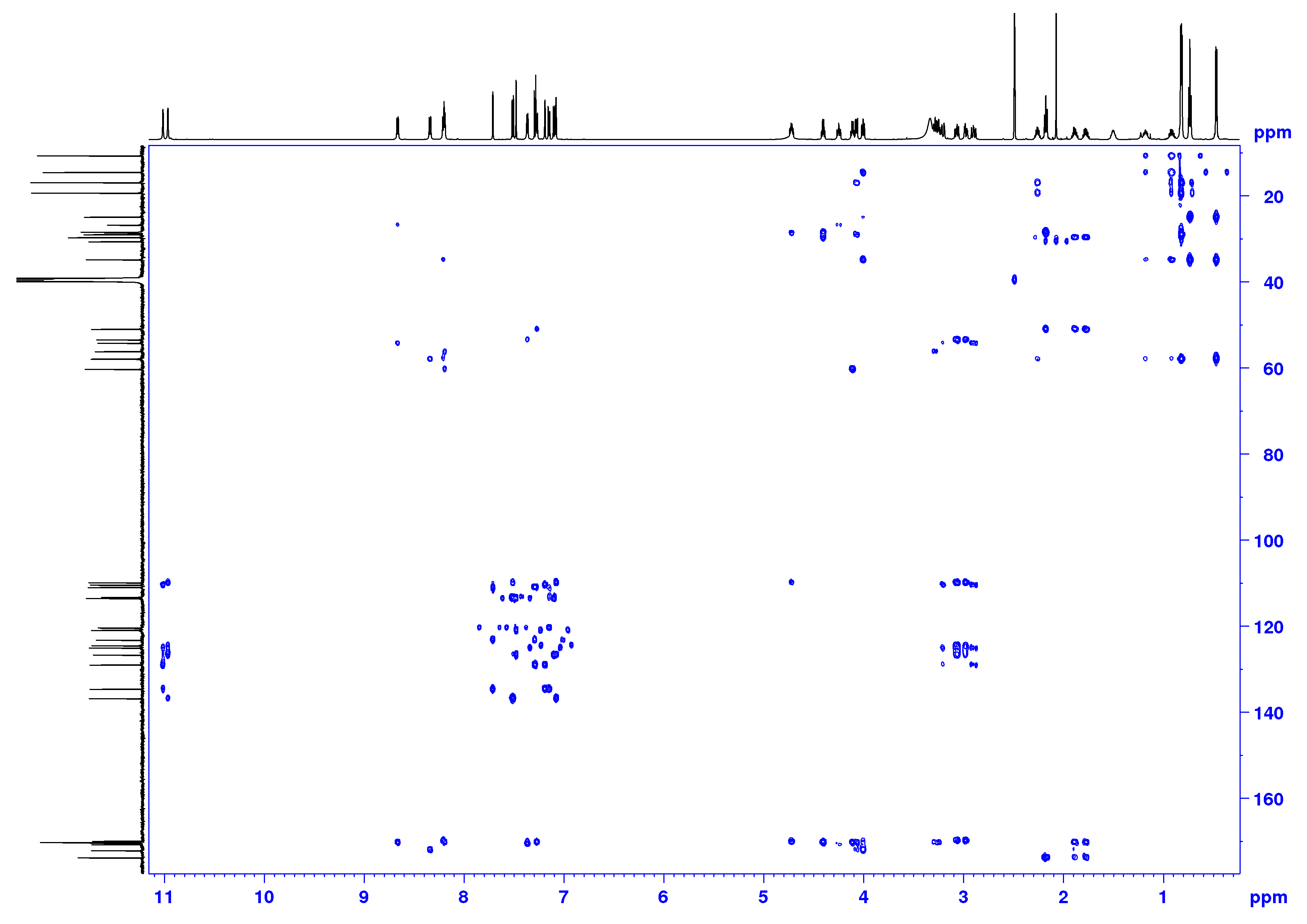


**Figure S18.** HMBC NMR spectrum (150 MHz, DMSO-*d*_6_) of suertide C (**3**).


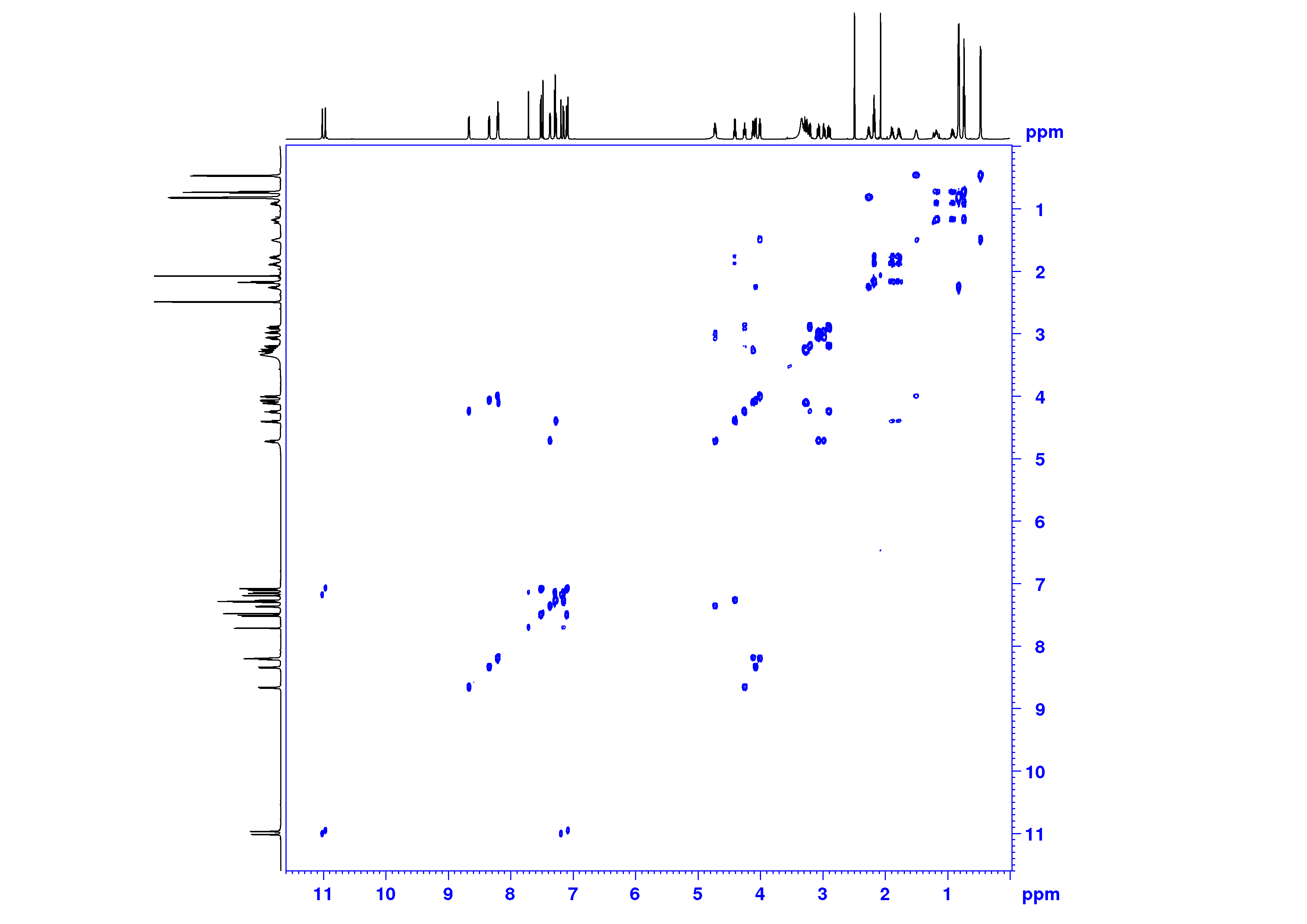


**Figure S19.** COSY NMR spectrum (600 MHz, DMSO-*d*_6_) of suertide C (**3**).


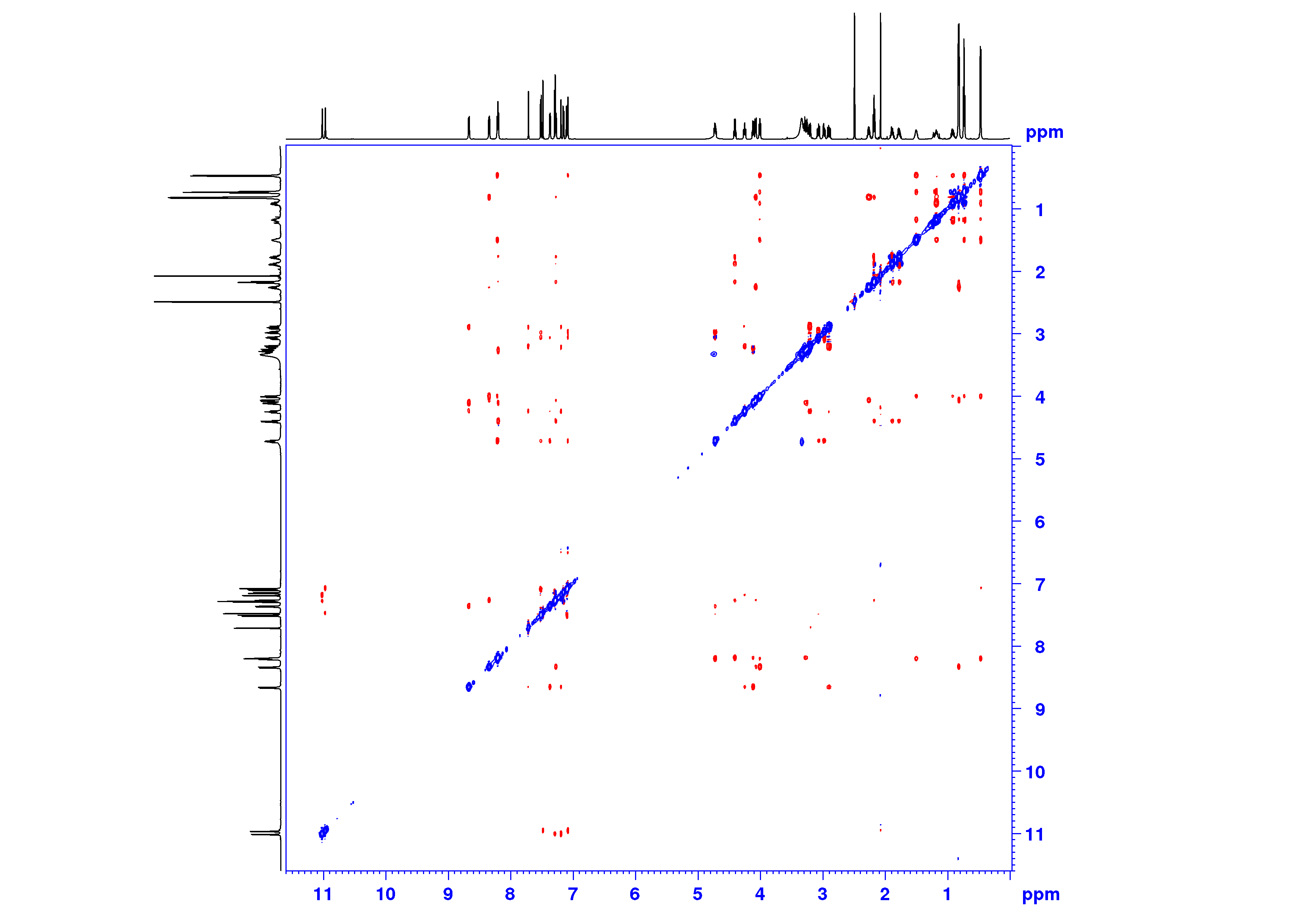


**Figure S20.** ROESY NMR spectrum (600 MHz, DMSO-*d*_6_) of suertide C (**3**).

| 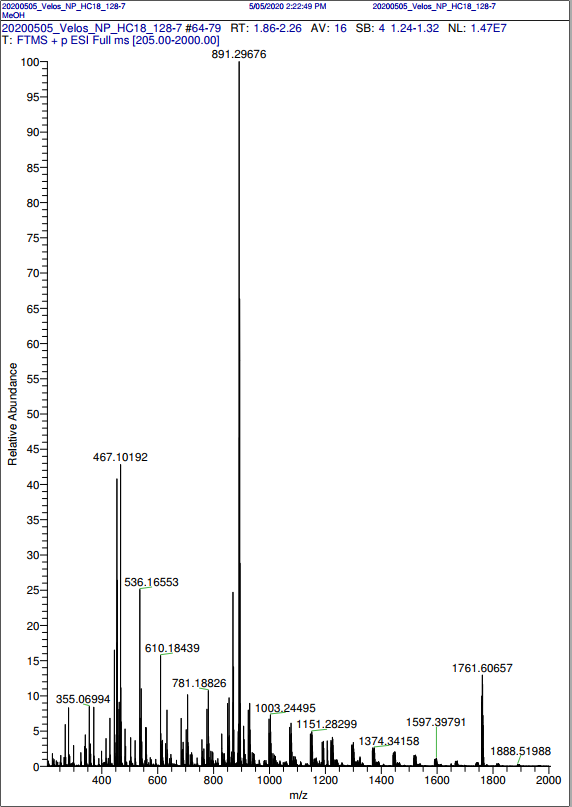 | 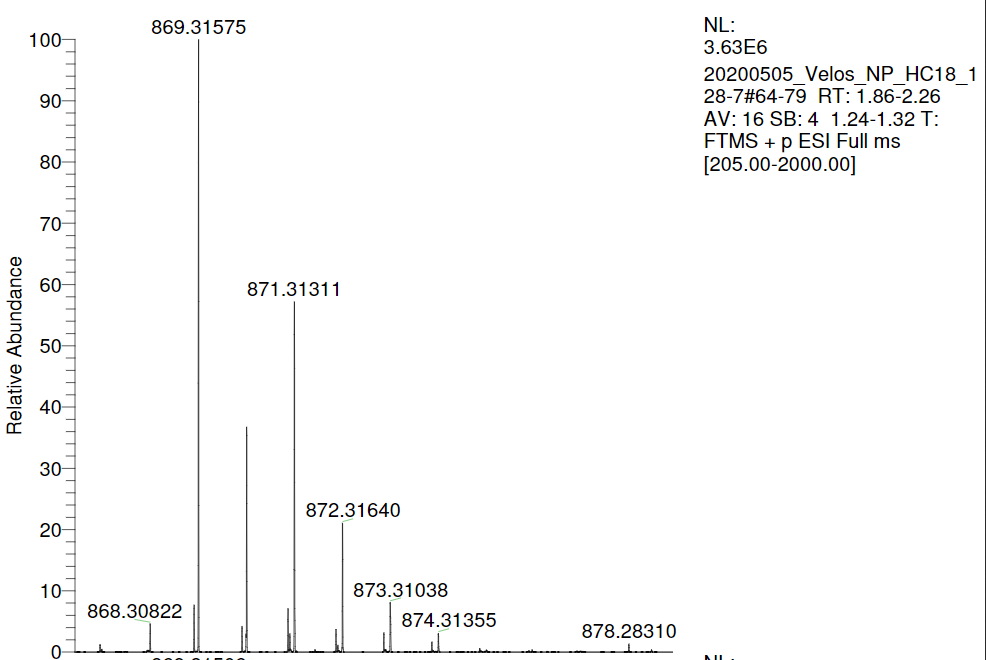 |
| --- | --- |

**Figure S21**. HRMS spectra of suertide A (**1**). Left: [M + Na]^+^. Right: [M + H]^+^.


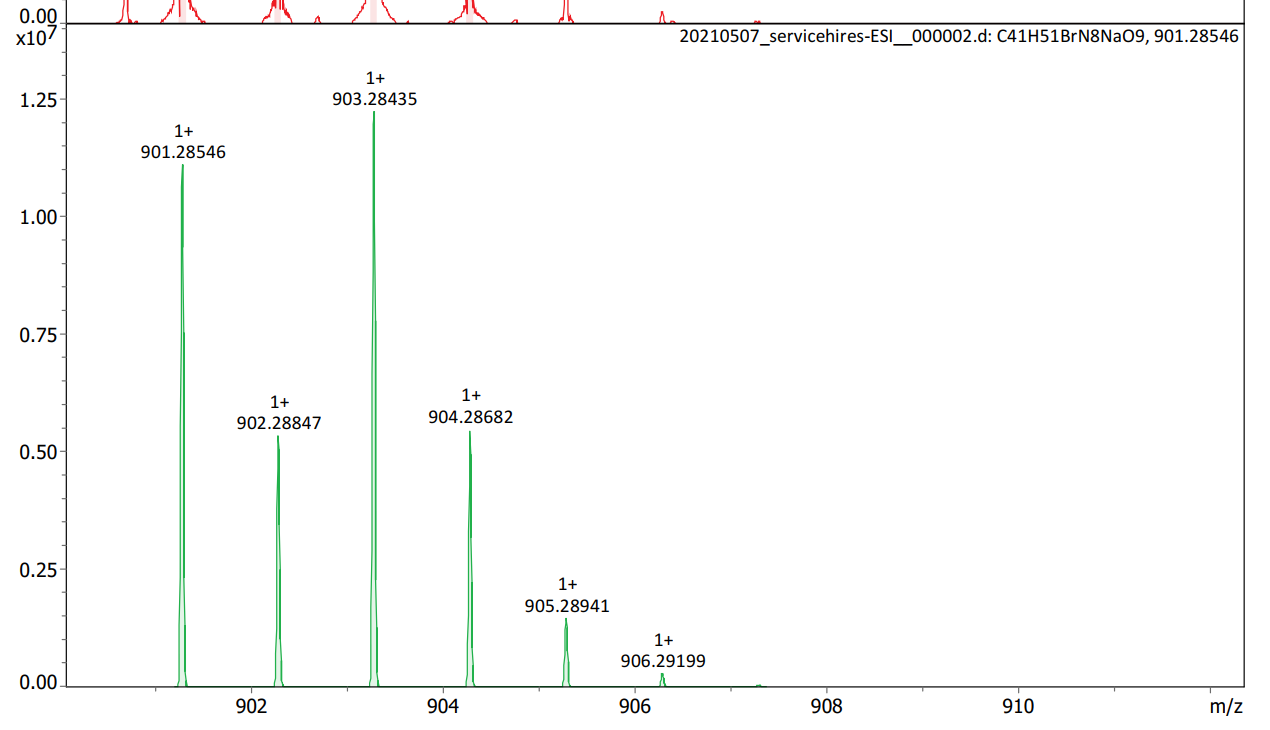


**Figure S22.** HRMS spectrum of suertide B (**2**).


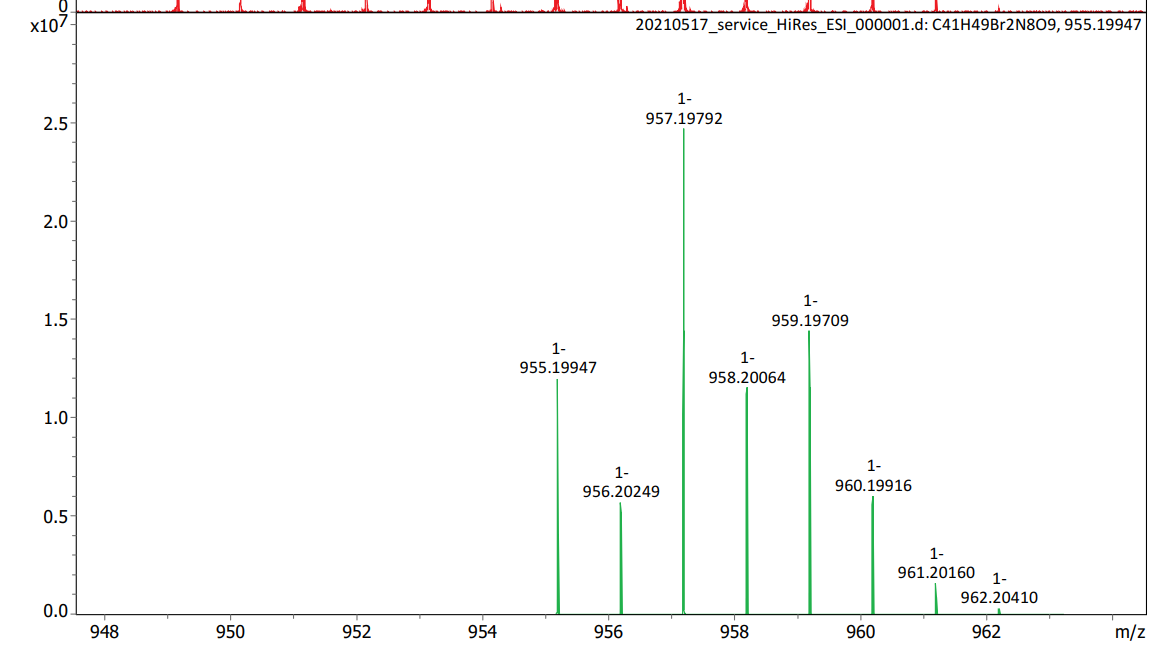


**Figure S23.** HRMS spectrum of suertide C (**3**).


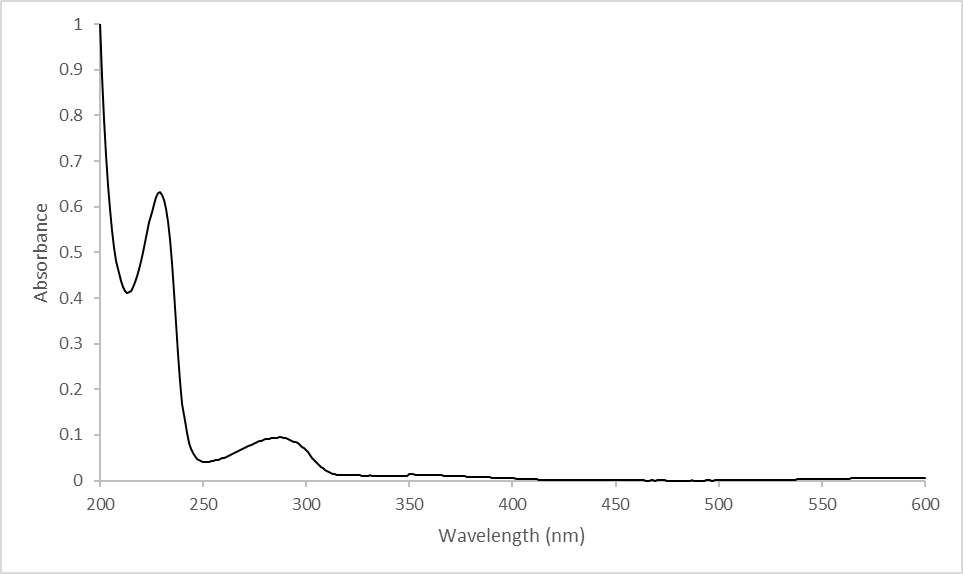


**Figure S24.** UV-vis spectrum of suertide A (**1**) in MeCN.


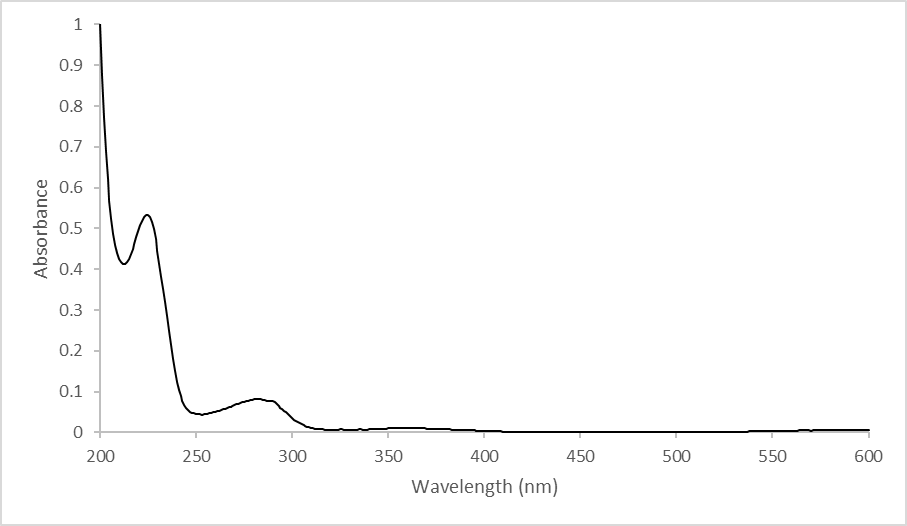


**Figure S25.** UV-vis spectrum of suertide B (**2**) in MeCN.


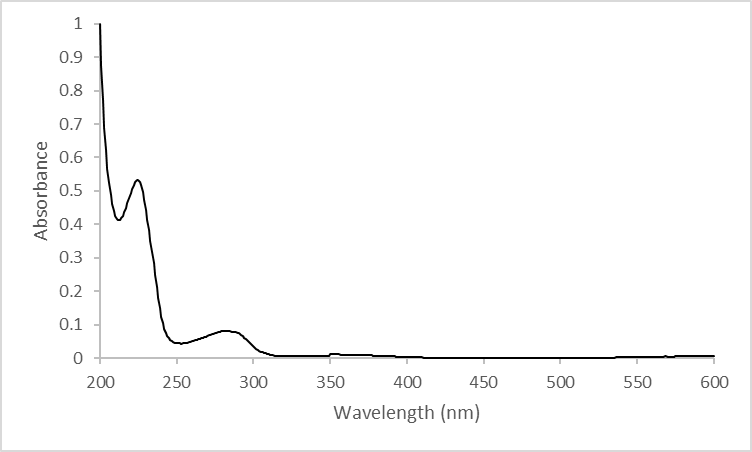


**Figure S26**. UV-vis spectrum of suertide C (**3**) in MeCN.

**Table S5.** The retention times and ions mass used to determine the absolute configuration of suertide A (**1**).

| Unit | Standard rt at 254 nm | Experimental rt (**1**) at 254 nm | *m/z* [M−H]^−^ |
| --- | --- | --- | --- |
| l-Ser | 3.89^a^ | - | 356 |
| d-Ser | 4.10^a^ | 4.07 | 356 |
| l-Glu | 4.30^a^ | - | 398 |
| d-Glu | 4.59^a^ | 4.63 | 398 |
| l-Val | 5.56^a^ | - | 368 |
| d-Val | 6.12^a^ | 6.12 | 368 |
| l-Ile | 6.04^a^, 5.29^b^ | 6.03^a^, 5.28^b^ | 382 |
| d-Ile | 6.59^a^ | - | 382 |
| l-*allo*-ile | 5.22^b^ | - | 382 |
| 5-Cl-l-Trp | 6.61^a^ | - | 489 |
| 5-Cl-d-Trp | 6.34^a^ | 6.61 | 489 |
| 6-Cl-l-Trp | 6.46^a^ |  | 489 |
| 6-Cl-d-Trp | 6.74^a^ | 6.74 | 489 |

^a^ C_18_ column; ^b^ C_3_ column


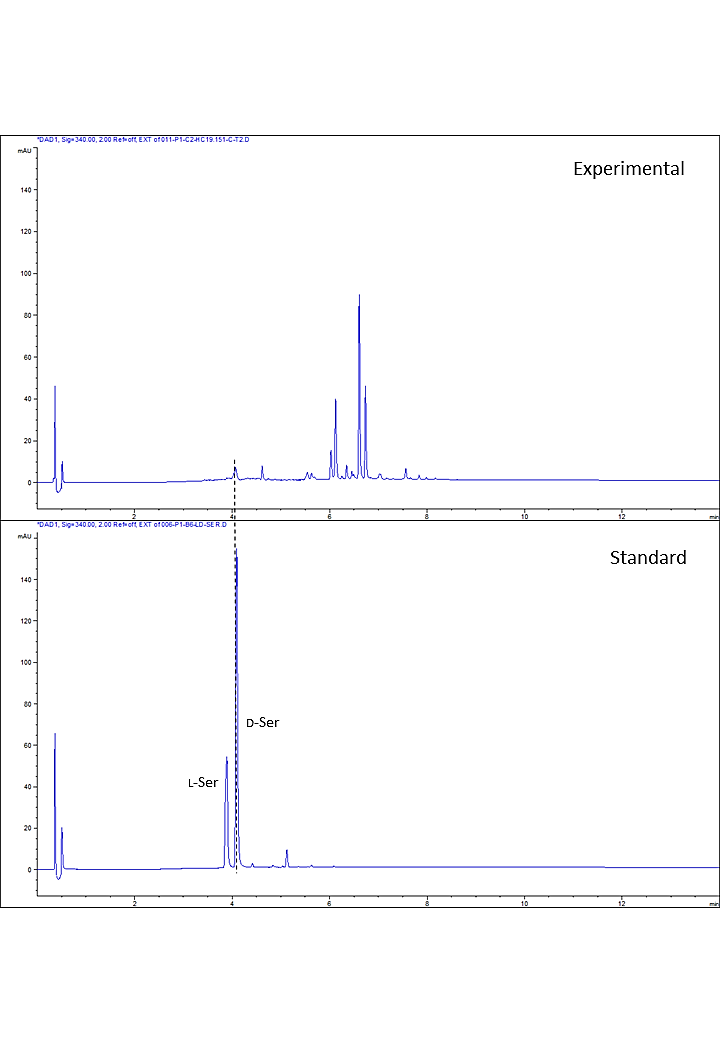


**Figure S27.** HPLC traces (340 nm) of hydrolysed suertide A (**1**) (top) and l- and d-serine (bottom) after derivatisation with Marfey’s reagent


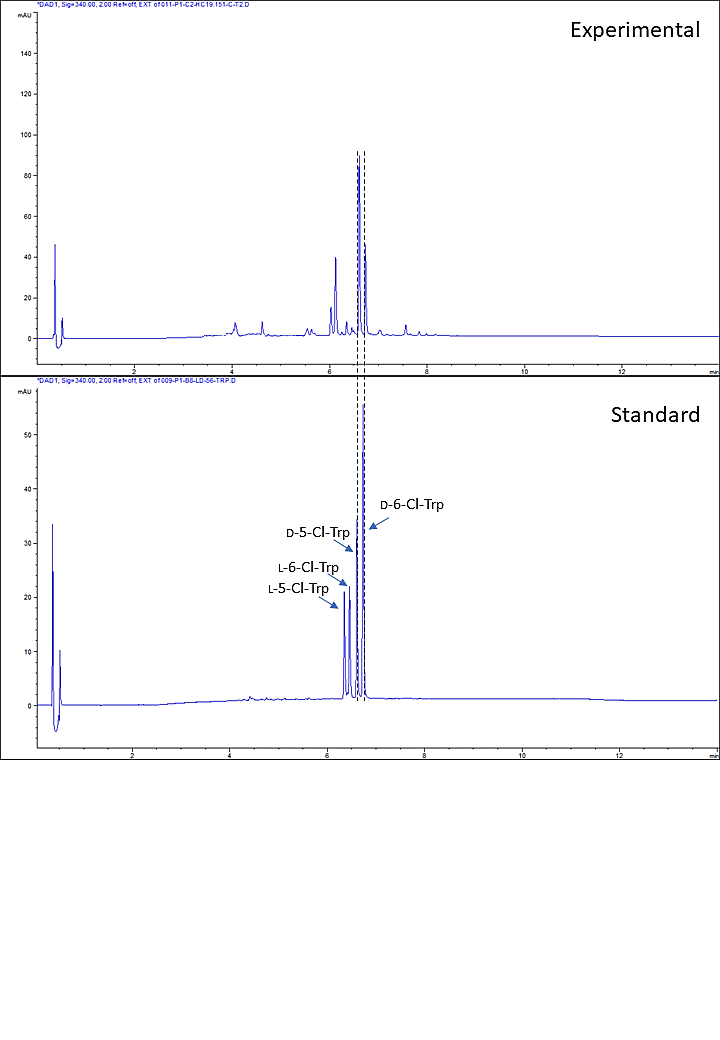


**Figure S28.** HPLC traces (340 nm) of hydrolysed suertide A (**1**) (top) and l- and d-, 5-Cl and 6-Cl-tryptophan (bottom) after derivatisation with Marfey’s reagent.


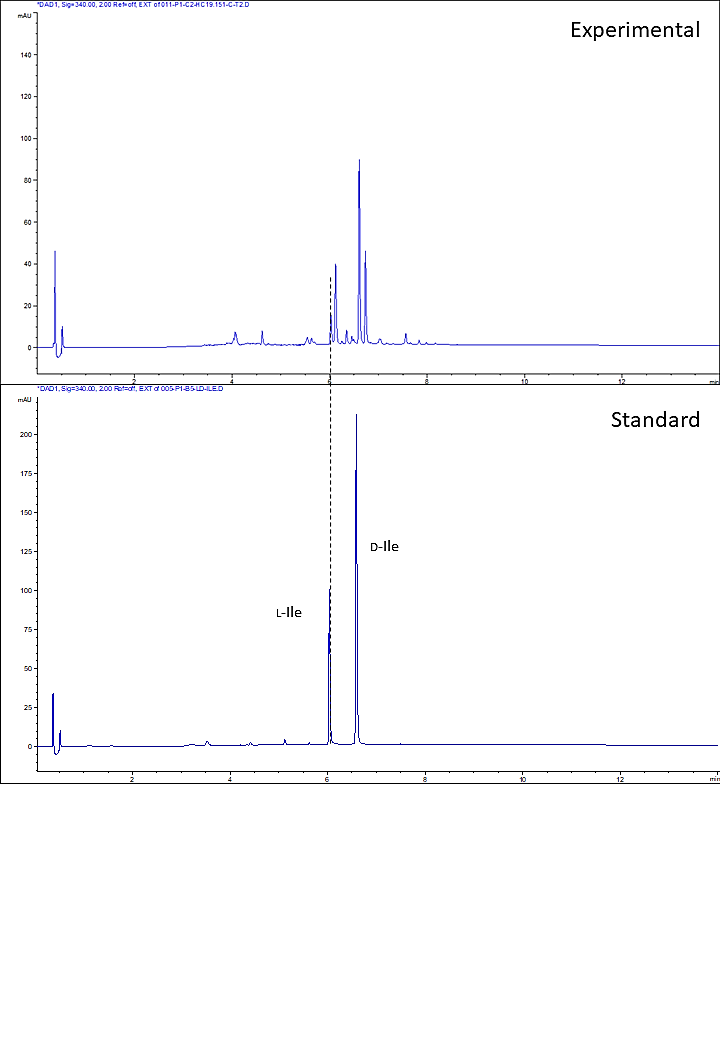


**Figure S29.** HPLC traces (340 nm) of hydrolysed suertide A (**1**) (top) and l- and d-isoleucine (bottom) after derivatisation with Marfey’s reagent.


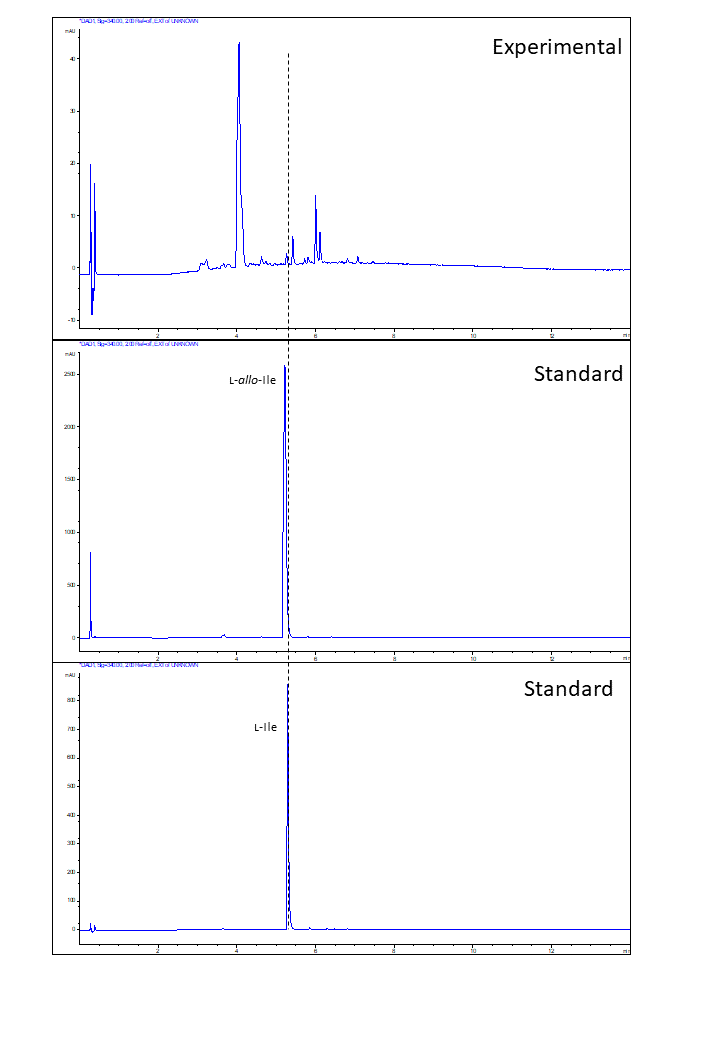


**Figure S30.** HPLC traces (340 nm) of hydrolysed suertide A (**1**) (top), l-*allo*-isoleucine (middle) and l-isoleucine (bottom) after derivatisation with Marfey’s reagent.


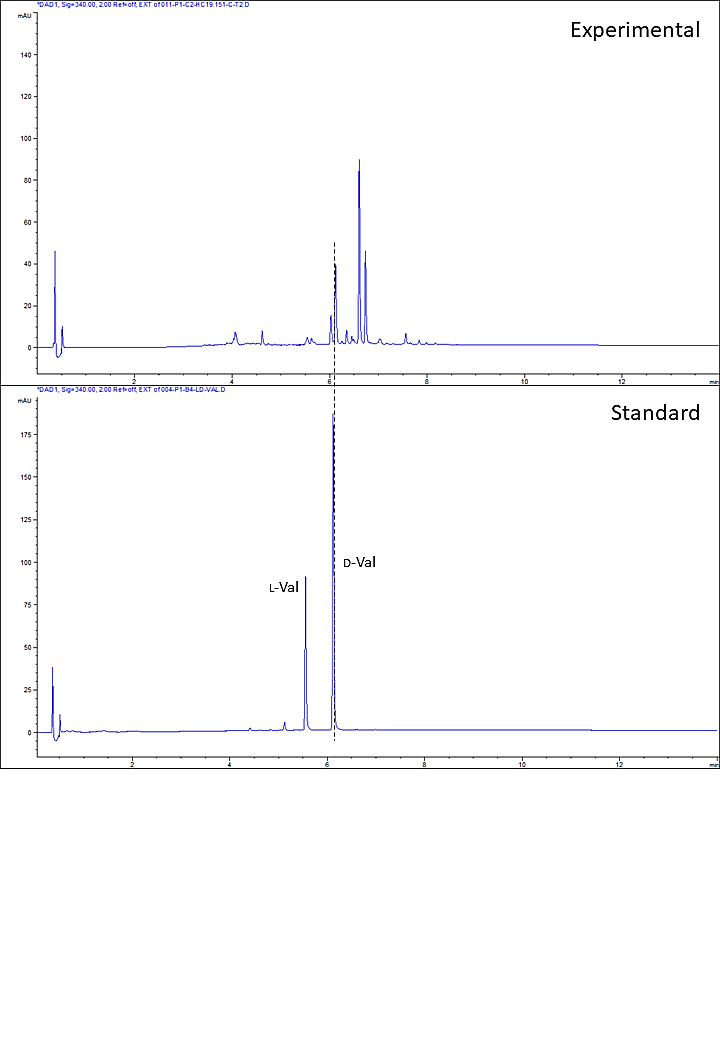


**Figure S31.** HPLC traces (340 nm) of hydrolysed suertide A (**1**) (top) and l- and d-valine (bottom) after derivatisation with Marfey’s reagent.


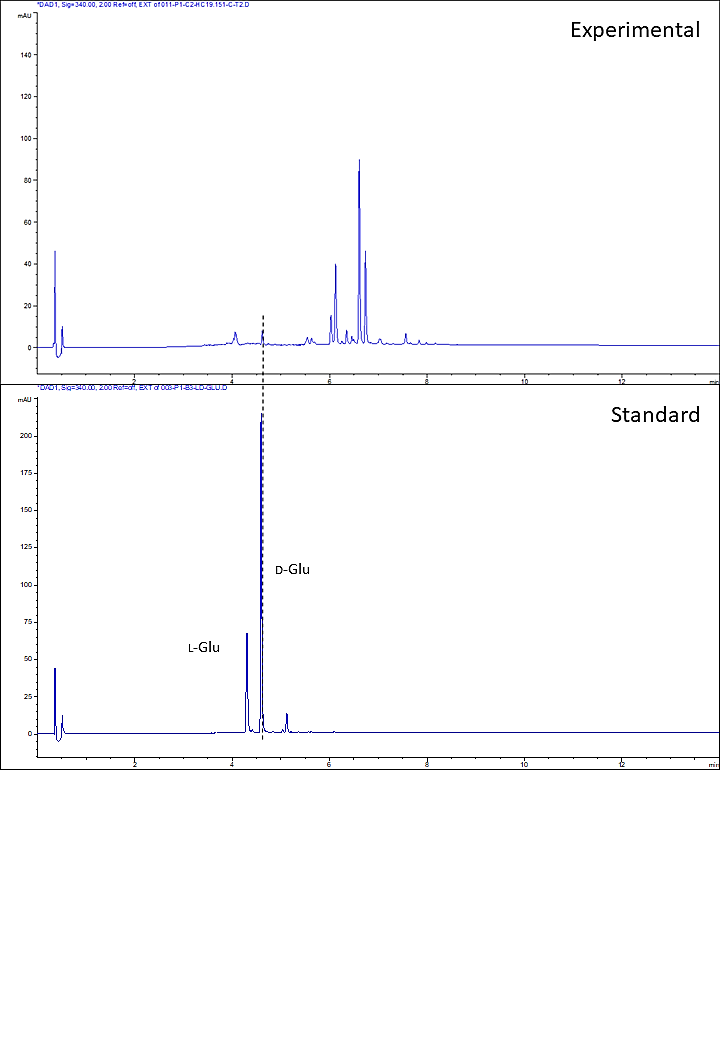


**Figure S32.** HPLC traces (340 nm) of hydrolysed suertide A (**1**) (top) and l-, d-glutamic acid (bottom) after derivatisation with Marfey’s reagent.


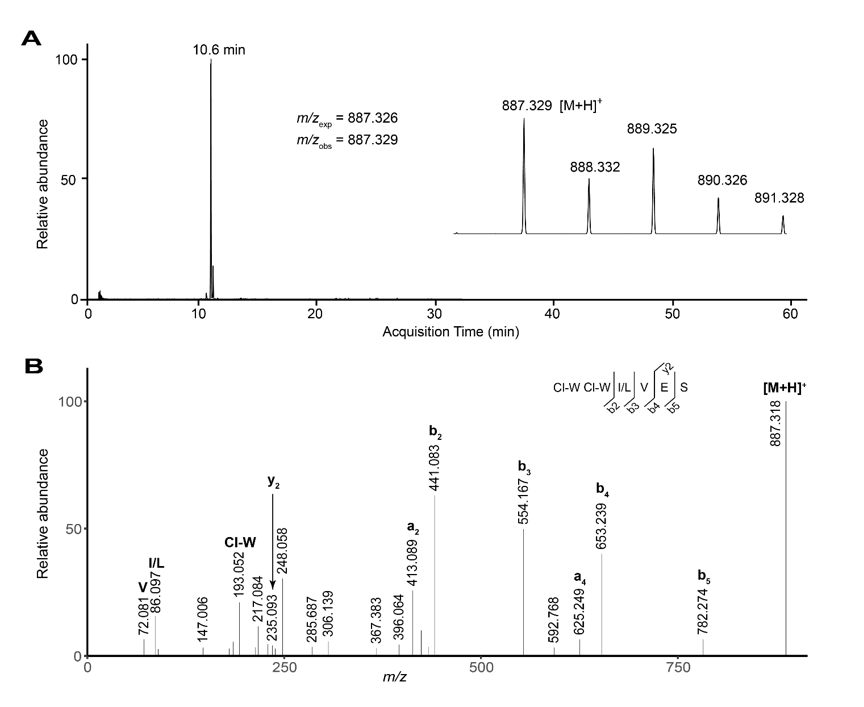


**Figure S33.** LC-MS data for suertide A (**1**) after linearisation with HCl.

Extracted ion chromatogram showing suertide A (**1**) peak at 10.9 min. Inset is the mass spectrum at the peak, showing the characteristic isotope profile for a chlorinated peptide*. m/z*_exp_ and *m/z*_obs_ are the expected and observed mass-to-charge ratios respectively. (B) MS/MS spectrum of the linearized peptide showing the amino acid sequence. Immonium ions are marked with the abbreviations of the associated amino acids. Cl-W is chlorotryptophan (the position of the Cl atom on the tryptophan indole ring cannot be determined). Leucine and isoleucine cannot be distinguished as they are isobaric.


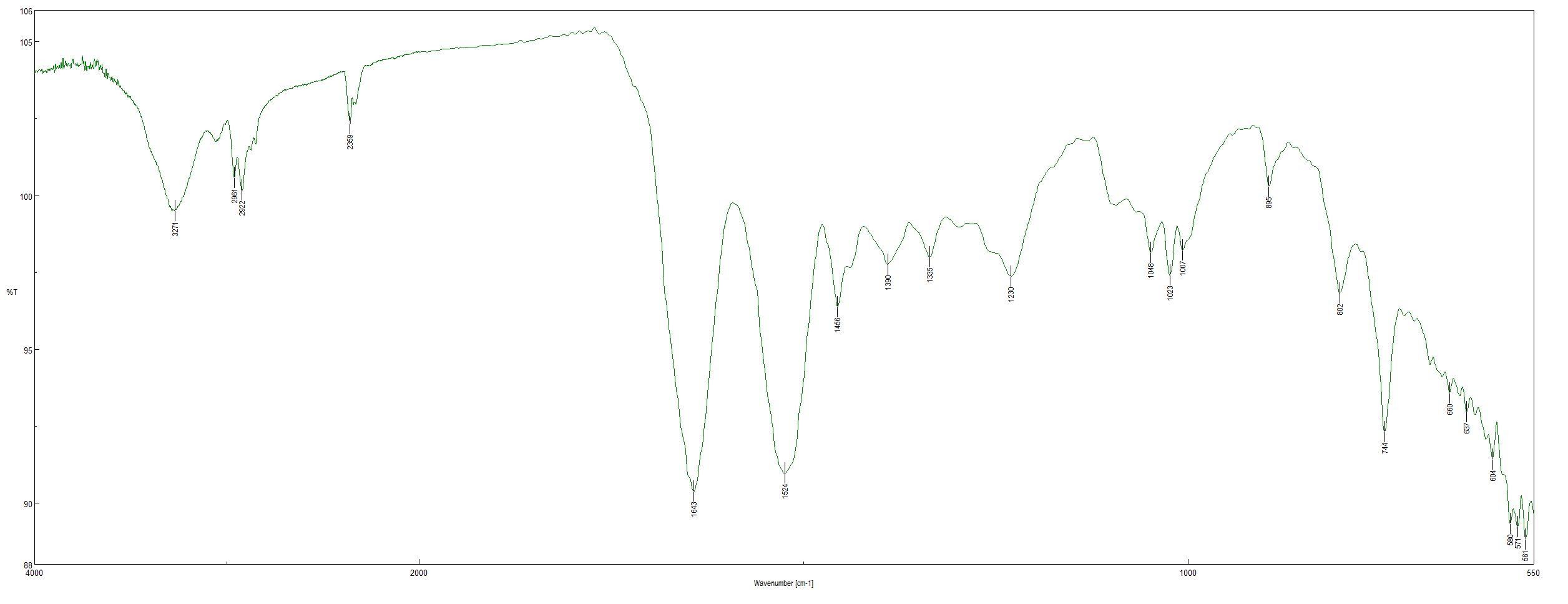


**Figure S34.** Infrared spectrum of suertide B (**2**).


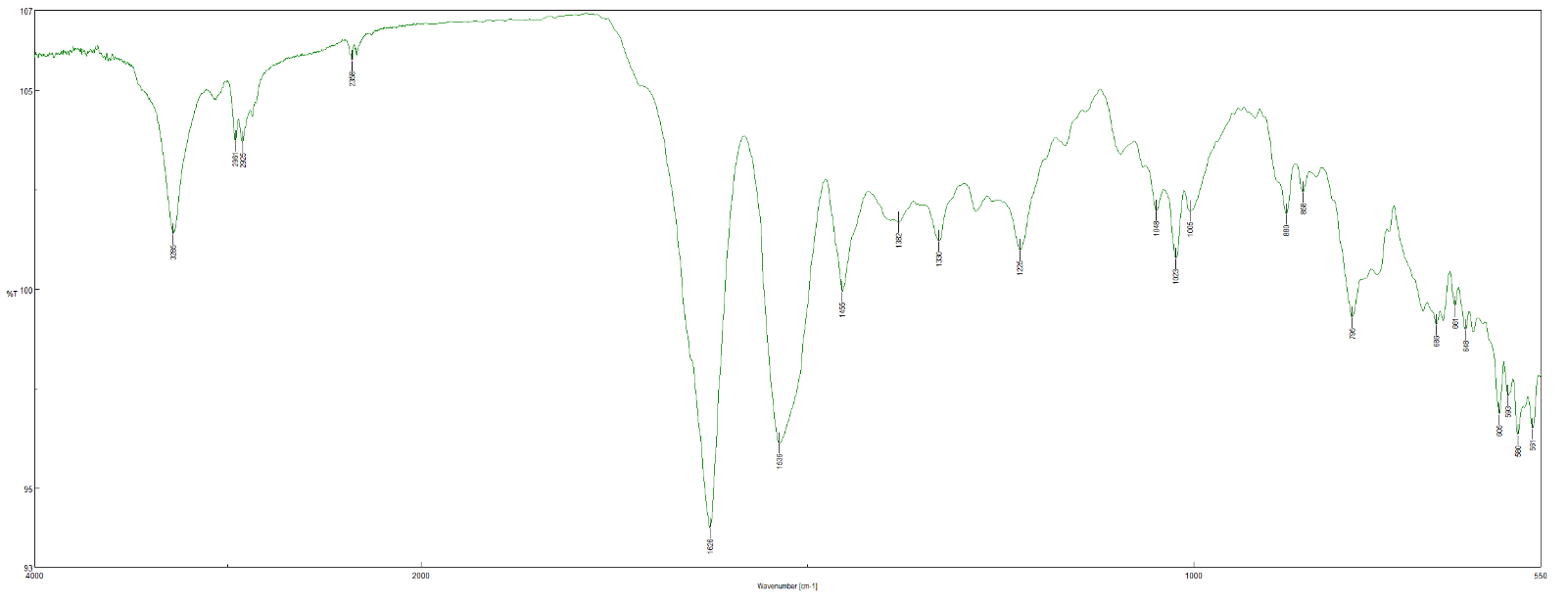


**Figure S35.** Infrared spectrum of suertide C (**3**).

# References

1 Lacey E.; Tennant S. Secondary metabolites: the focus of biodiscovery and perhaps the key to unlocking new depths in taxonomy. *Microbiol Aust.* 2003; 24: 34-35.
